# Force production parameters as behavioural measures for anger expression and control: The Method of Stamp Strike Shout

**DOI:** 10.1371/journal.pone.0206494

**Published:** 2018-11-14

**Authors:** Cees Boerhout, Jooske T. van Busschbach, S. Marije Vermerris, Nadine A. C. Troquete, At L. Hof, Hans W. Hoek

**Affiliations:** 1 University Center of Psychiatry, University Medical Center Groningen, University of Groningen, Groningen, the Netherlands; 2 Lentis Psychiatric Institute, Groningen, The Netherlands; 3 Department of Human Movement and Education, Windesheim University of Applied Sciences, Zwolle, The Netherlands; 4 Center for Human Movement Sciences, University Medical Center Groningen, University of Groningen, Groningen, The Netherlands; 5 Parnassia Psychiatric Institute, The Hague, The Netherlands; 6 Department of Epidemiology, Mailman School of Public Health, Columbia University, New York, NY, United States of America; Universita degli Studi di Padova, ITALY

## Abstract

This study presents first test results of a new performance-based, psychomotor method to measure anger expression and control, based on voice expression and physical force production in directional movement of arms and legs, called the Method of Stamp Strike Shout (MSSS). Recorded are the standardized impact of stamping on a force plate, hitting a punching bag, and the amplitude of shouting in a microphone at various force levels. The premise is, that these body behaviours stand for the ‘urge to act or shout’ that belongs to anger-related emotions. The MSSS is meant to be applied in addition to potentially biased self-report questionnaires and has been designed for diagnostic as well as therapeutic purposes in clinical practice. First, this paper focusses on the instrumentation, internal structure and reliability of the MSSS. An explorative study in a student sample (n = 104) shows correlation patterns between increasing and decreasing levels of force production within each subtest (Stamp, Strike and Shout) and between the three subtests. We found excellent internal consistency of the three subtests and high test-retest reliability. The parameters of increasing and decreasing force levels form the slopes of what we call a force pyramid. To adjust for the clustering within persons, aggregated outcomes were calculated: sum scores per subtest as an indication of total force produced, two linear contrast scores to indicate the rate of increase / decrease, and two quadratic contrast scores as measures of the curvature of the slopes. On all subtests, all aggregated scores showed differences between men and women, also when controlled for weight. To test the validity of the MSSS, the second part of the paper examines the relationship between force parameters and anger coping style, measured by the Self-Expression and Control Scale (SECS). The results suggest that the Shout subtest was the most sensitive indicator for anger coping style, showing negative correlations with Anger In, for women as well as men. For women, higher amplitude was also associated with higher Anger Out and lower amplitude with higher Anger Control. The Stamp subtest showed weak positive correlations with the Anger In subscales, whereas no correlations were found on the Strike subtest. Further, a more robust comparison was made between two groups of participants who reported to have an internalizing versus an externalizing anger coping style. Results indicated that internalizing women as well as men used less force than externalizing participants on all three subtests, especially on the Shout subtest. This was confirmed by lower mean sum scores on the Shout subtest for internalizing women compared with externalizing women. No differences in linear contrast scores were shown between internalizing and externalizing participants. The quadratic contrast scores suggested differences of the curvation of the slopes between women with more or less anger control when stamping, and men with more or less anger control when striking. As this is an explorative study, findings should be interpreted with caution.

## Introduction

Notwithstanding the importance of emotions as an outcome measure for psychological functioning, a long history of psychometric research has shown that developing instruments to quantify a person’s emotional state still confronts researchers with methodological challenges in realizing ecologically valid measures. Despite adaptations, self-report measures remain limited by response biases and are not telling the whole truth [1]. Additional performance-based measures are needed to estimate behavioural and non-verbal aspects of emotion expression. Observational methods in laboratory or real-life situations may be a next step towards ecologically valid measurement of personal characteristics influencing the expression of emotion.

In their review of measures of emotion, Mauss and Robinson [2] start from a consensual model that distinguishes three levels of emotional responses: subjective experience, physiological reactions and subsequent behaviour. The behavioural level refers to theories that infer emotional states from vocal characteristics, facial displays, and body behaviours. These theories are based on linking emotions to communicative functions [3] or to action dispositions, like the tendency to fight or flight [4]. More research needs to shed light on behavioural measures of emotions. The present study focuses on measurement of body behaviour and vocal characteristics to contribute to a valid assessment procedure of anger coping. The ability to produce and to regulate physical force is studied in relation to the degree to which someone regulates anger expression or inhibition.

Research on body behaviour (movement, expression, posture) as measure of emotion is relatively sparse [2], despite the importance of non-verbal communication [5]. On the level of motor behaviour research indicates that pleasant and unpleasant emotions modulate force production [6–8]. Anger may facilitate physical performance, depending on the demands of the task, but findings point towards a complex role of individual differences in the anger-performance relationship and emotion regulation [9]. However, compared with fear, sadness and happiness, anger remains relatively understudied (in terms of neuronal and physiological mechanisms of action) and it is harder to predict the likely influences of anger on cognition and behaviour [10].

Regarding the influence of emotions on vocal characteristics, the basic assumption is that measurable voice parameters reflect a person’s affective state. The physiological reactions involved modify the voice production process [11,12]. Sympathetic arousal associated with anger often produces changes in respiration and muscle tension, which influence the acoustic characteristics of speech [11]. The most common measures are voice amplitude (loudness) and pitch (fundamental frequency). High-arousal emotions like fear, anger, and joy are linked with higher pitch than lower-arousal emotions such as sadness [13]. It is more difficult to find vocal characteristics that are linked to valence. Anger and joy are similar in arousal, but different in valence, yet both emotions have been linked to comparable vocal pitch and amplitude [14].

This paper introduces first test results of a custom-made performance-based measuring method for anger and aggression based on physical force production in directional movement of arms and legs and in voice expression. Recorded are the momentum of stamping on a force plate and hitting a punching bag, and the amplitude of shouting in a microphone. The method is called the ‘Method of Stamp Strike Shout’ (MSSS). Levels of force production and force control are expected to serve as an indication of anger and aggression regulation. The MSSS is meant to be used in addition to self-report methods of measuring anger and aggression. These methods reflect one’s retrospective perception of emotional responding rather than the emotional response itself and may be biased by social desirability, denial, and awareness deficits, as has been reported in case of anger [15]. Real-time assessment of body behaviour and vocal characteristics may add to ecologically valid measurement.

The rationale for the construct of the MSSS has been inspired by the temper tantrum of toddlers uncontrollably waving their arms and legs and screaming at high decibels as a result of the adrenaline rush. Apparently, the ‘urge to act and shout’ which belongs to anger primary finds an outlet in expressive movement by arms and legs and by voice expression (or breath holding spell). Physical responses like clenched fists, tense muscles, and swallow breathing belong to the trigger stage of the anger assault circle [16]. The MSSS offers the opportunity to observe the body in action and to combine quantitative outcome measures with qualitative observation and post-test interview.

The MSSS has its origin in psychomotor therapy (PMT), a body and movement-oriented therapy that is well integrated in mental health care in the Netherlands and Belgium [17]. PMT integrates body experiences and cognitive-emotional functioning in approaching aggression regulation in psychiatric patients. The idea of the MSSS emerged from PMT in the field of eating disorders. Eating disorders are often undetected, although worldwide epidemiological studies show high prevalence rates especially among girls and young women [18]. Within this field there is an active search for the development of new treatments for eating disorders and related psychopathology [19]. PMT for eating disorders targets on persistent anger issues by enabling patients to practice body expression including force production exercises such as used in the MSSS. Voice and movement exercises in PMT were found to be effective in the treatment of excessive anger inhibition in patients with eating disorders [20,21]. The MSSS is meant for diagnostic as well as therapeutic purposes. We developed the instrument at the Center for Human Movement Sciences in cooperation with the Technical Support Unit of the Faculty of Science and Engineering, at the University of Groningen in The Netherlands. The instrument consist of three subtests:

STAMP–For the Stamp subtest, a simple portable force plate with force transducer measures vertical forces generated by stamping.

STRIKE–For the Strike subtest various methods were available, for example: using force sensors inserted into a target-block mounted on a lath [22], measuring acceleration of a pendulum arm [23], and using a dummy (head, neck, and torso) to measure punch velocity and force [24]. Other potential measures are a power sensor unit to be placed into the boxing glove instead of mounting it on the target [25,26], or into a wristwatch device [27], and a flexible impact force sensor on a load cell, a concrete wall or a sandbag [28]. For the MSSS an accelerometer embedded in a punching bag was chosen. The use of a bag fits well with our therapy practice when working on aggression regulation issues. In a recent study the use of an accelerometer in a bag showed a small measurement error [29].

SHOUT–For the Shout subtest, a microphone at a fixed distance recorded the amplitude of voice expression.

### Testing the Method of Stamp Strike Shout

The objective of our study is to test the reliability and validity of the MSSS in a non-clinical sample of 56 women and 48 men in a laboratory set-up. First, we introduce the instrumentation and task. Then, an explorative study focusses on the internal structure of the MSSS by measuring the intra-test correlations between four intensity levels of force production per subtest (25-50-75-100% maximum force) and by determining the inter-test correlations between the three subtests (Stamp, Strike and Shout). Within-subject variations and test-retest correlations indicate the reliability of the MSSS: the degree of precision and reproducibility of the routine.

To test the validity of the MSSS we studied whether and how levels of physical force production are associated with self-reported levels of anger expression and control. It may be tempting to hypothesize that someone’s propensity to express versus inhibit anger feelings results in higher versus lower force production. However, it may as well be hypothesized that someone who is used to bottle up anger, or is prone to inhibit verbal expression, is the more triggered to engage in non-verbal expression, particularly when invited to produce 100% force. As the anger-performance relationship is complex, depending on individual differences in anger experience, anger regulation style and personality traits [9], the direction of the correlations is hard to predict. Since, for now, the contours of such a convergent system are not yet clear, our study has an explorative character with a large number of associations to be assessed and we will refrain from confirmative hypothesis testing.

The objective is to explore whether and how the three subtests, Stamp, Strike and Shout, converge into a coherent response system that can be used for clinical and research purposes. To address our research question we analysed whether and how performance on the three subtests of the MSSS (a) correlates with self-reported degrees of anger internalization, anger externalization, and anger control, and (b) differs between two groups of participants respectively showing an internalizing versus an externalizing anger coping style.

## Methods

### Participants

**Fig 1:**
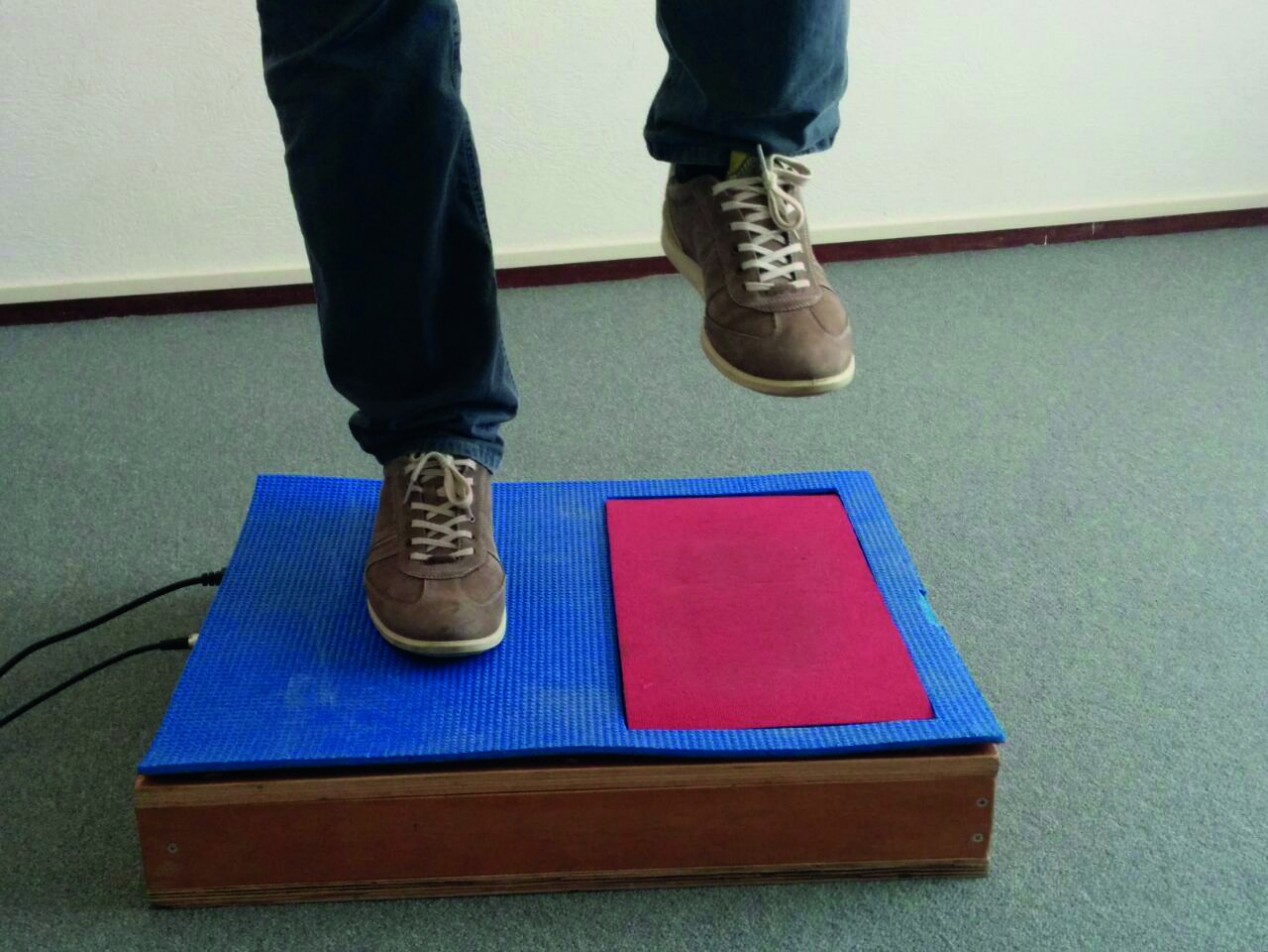
Stamping on the force-sensitive area of the force plate.

**Fig 2:**
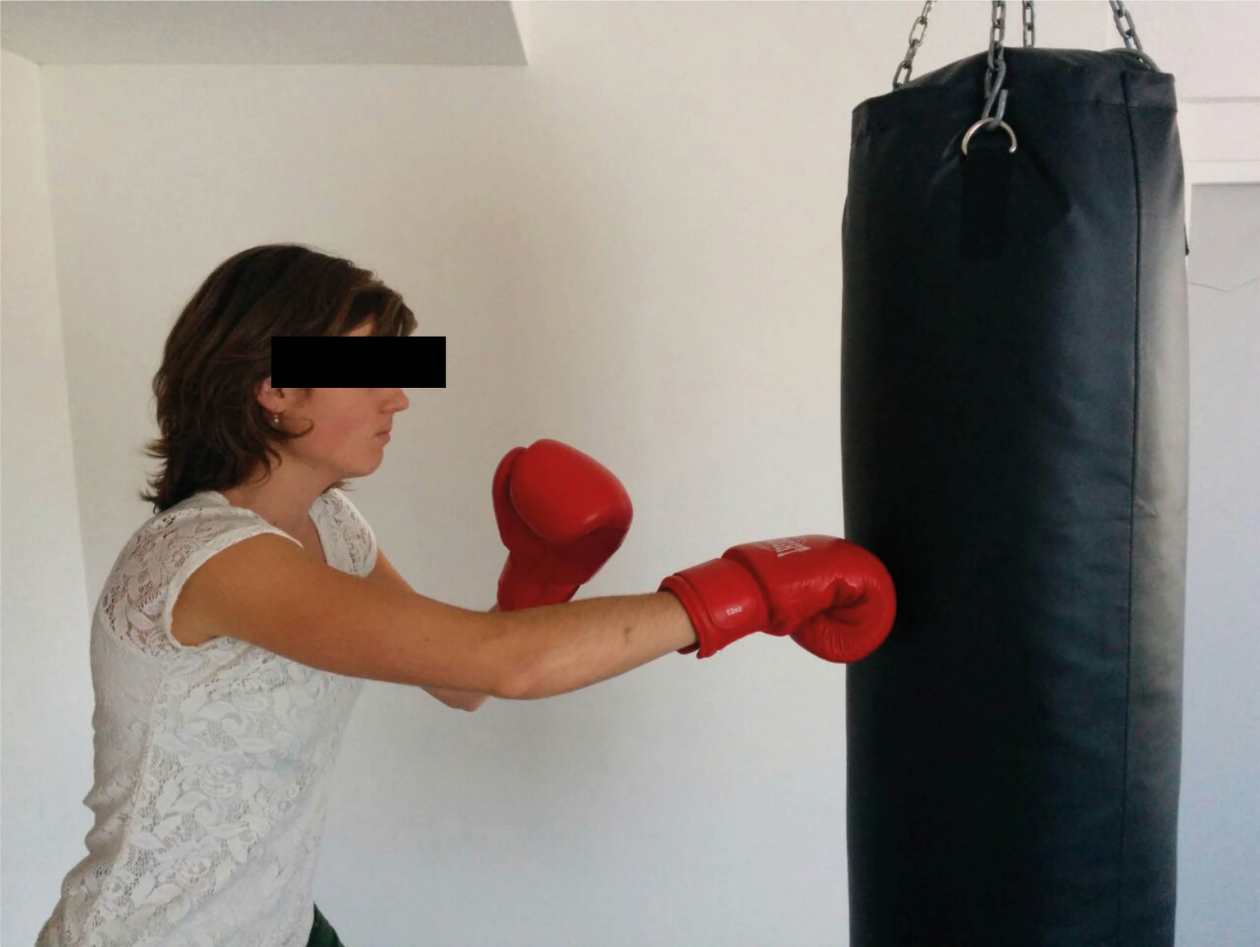
Striking the mid-section of a bag.

**Fig 3:**
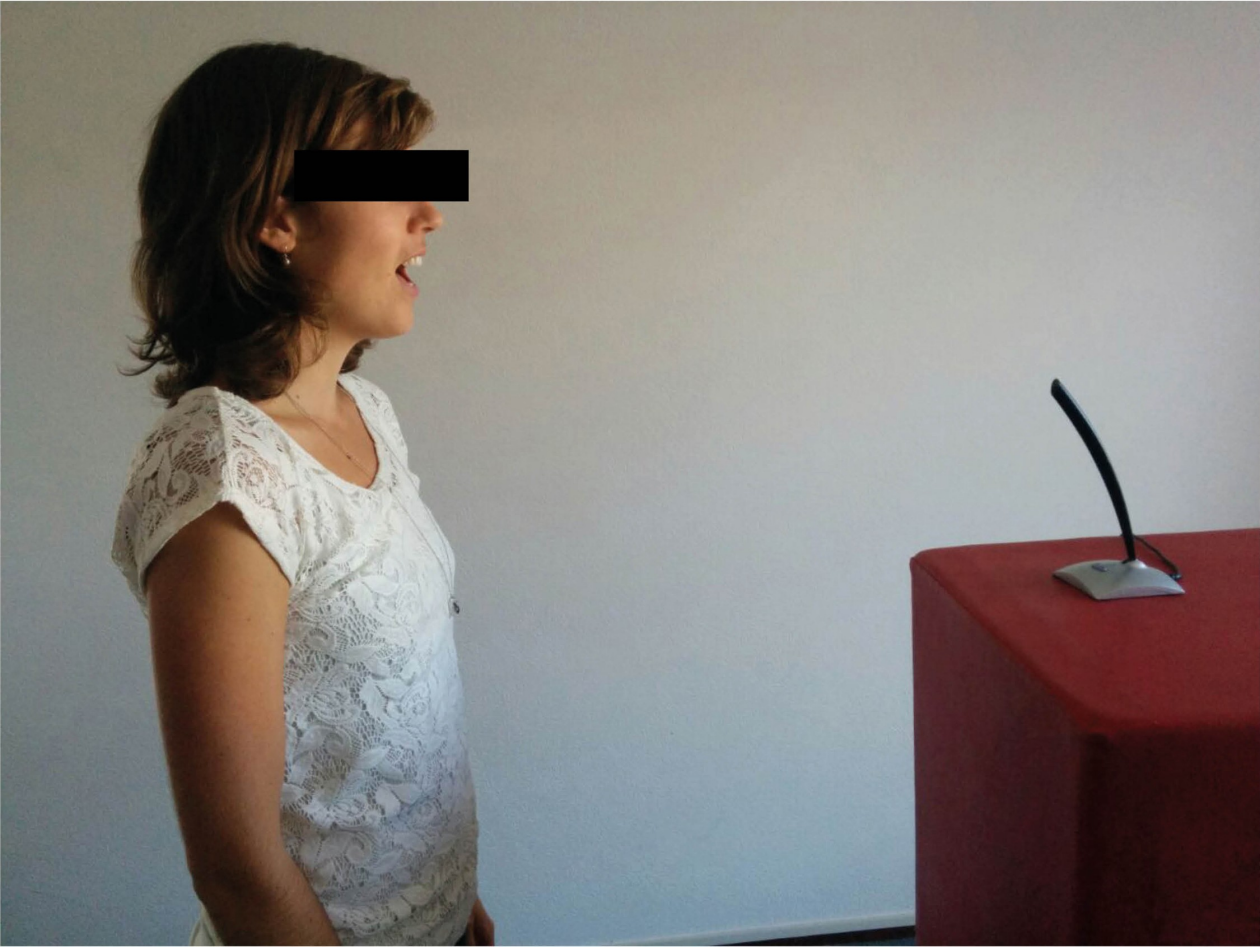
Shouting ‘Haa’ in a microphone.

One hundred and four students (48 men, 56 women; M_age_ = 20.84, SD = 2.28), recruited through undergraduate courses and personal networks, volunteered for this study. Exclusion criteria were: (a) injuries to wrist, arm, shoulder, foot, leg, hip and a sore throat; and (b) using tranquilizing medication. Institutional ethics approval was gained (Center for Human Movement Sciences, University Medical Center Groningen, University of Groningen, The Netherlands). The researcher informed potential participants briefly about the study objective: to measure force production and to relate the results to the outcome of a questionnaire on anger coping, after which participants were asked to sign an informed consent. All enrolled participants completed a questionnaire on personal characteristics to check for possible confounders, i.c. body weight, trained vocal skills, boxing experience. Then they received explanation about the test procedure. Out of 104 participants 22 were tested twice with an interval of five months. Only these 22 received a financial compensation for their participation. Informed consent was also signed for publishing the images of the individual pictured in Figs 1–3.

### Procedure

We tested the MSSS in a practice laboratory at the Center for Human Movement Sciences, University of Groningen, The Netherlands. Prior to the test participants completed questionnaires on demographics, personal characteristics, and anger coping. Actual weight and body length were measured. Thereafter, participants performed predefined light exercises to stretch and warm up their muscles and prepare for the test. The researcher provided technical instruction on how to perform the three subtests. A randomisation procedure determined the sequence of the subtests. The participants drew a note with one of three possible sequences out of an envelope. After performing the subtests the researcher checked whether the participant had experienced emotional arousal instigated by the test. If needed, the researcher could help to reduce tension at the end of the procedure.

### Task

After standardized randomisation of the sequence, participants performed the three different subtests of the MSSS: stamping, striking and shouting. They executed the three subtests twice, with increasing and decreasing force. The instruction was to produce respectively 25%, 50%, 75% and 100% of their maximum force, and then back to 75%, 50% and 25%. This sequence represents a *force pyramid* used to quantify one’s ability to produce and control physical force. The test was self-paced with no instructions about the timing, maximum force or feedback on performance.

During the Stamp subtest, the participants stamped a sequence with one foot at the time on a force plate (Fig 1), wearing shoes with flat soles. At every force level the participant stamped four times with each foot. The instruction was to lift de foot directly after stamping. In the Strike subtest, participants stroke a boxing bag four times at every force level, alternating with the left and right hand with both hands in gloves (Fig 2). They alternated between left and right hands and feet to control for the extra power and possible better regulation through the dominant side [30]. In the Shout subtest, the participants shouted ‘Haa’ in a microphone (Fig 3), once at every amplitude level. The scores on the two repetitions of each subtest were averaged in order to achieve an acceptable level of measurement error [31]. The overall duration of the task was approximately 25 minutes.

### Instrumentation

**Fig 4:**
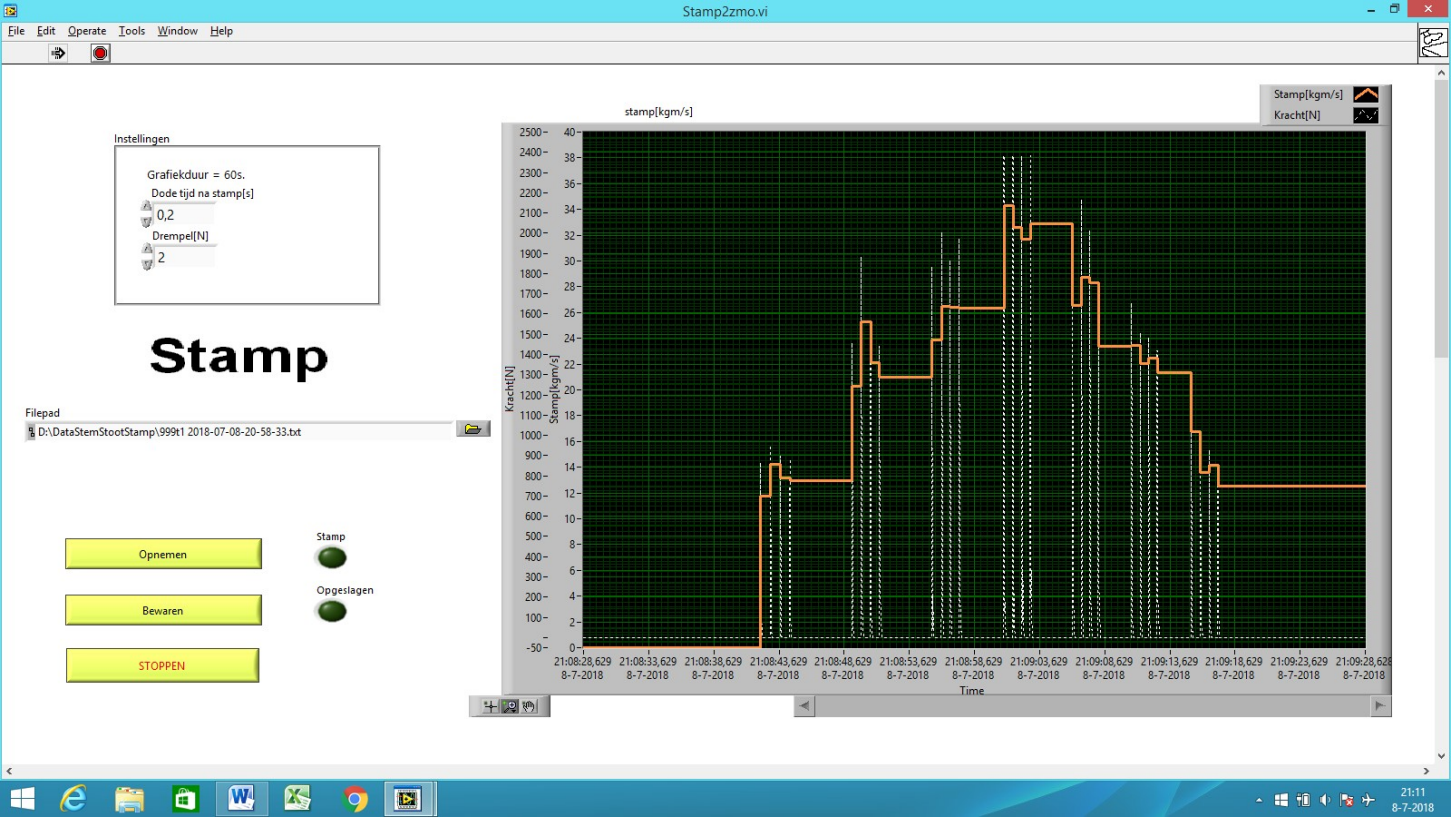
LabVIEW image of the Stamp subtest.

A custom-made LabVIEW program controls all three subtests of the MSSS (Fig 4). The output is a text file with the summary score of every stamp, strike or shout in the course of the trials. Sampling of analogue signals in the stamp and strike mode is done by an A/D converter USB-NI6008 (National Instruments).

#### STAMP

**Fig 5:**
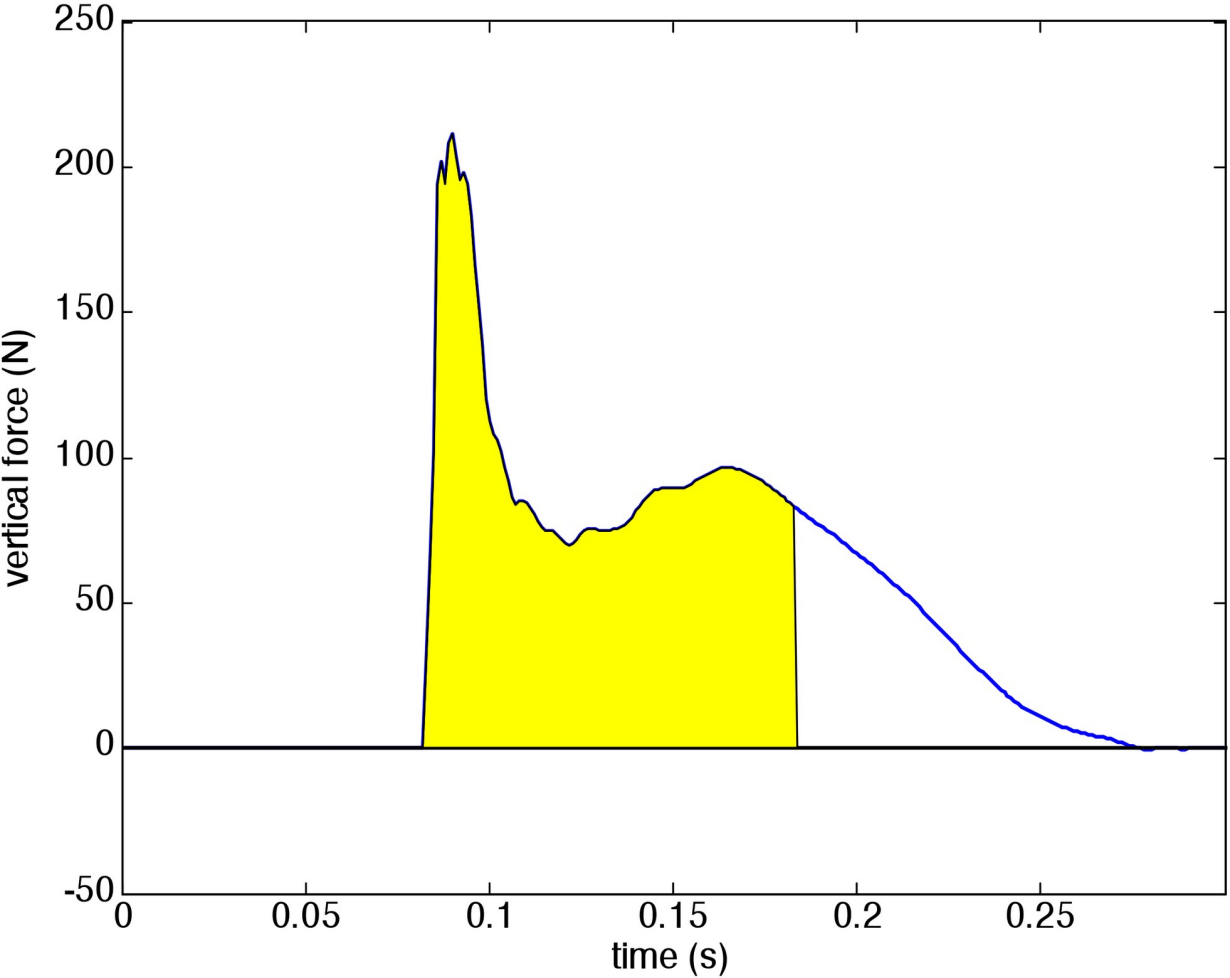
The low-pass filtered force signal of a stamp. The impulse of the foot stamp is equal to the shaded area under the curve $p_{stamp}=\int_{t_{1}}^{t_{2}}Fdt$, with *t*_*1*_ 10 ms before the threshold (2 N) is passed and *t*_*2*_ 100 ms after the threshold crossing.

The ‘stamp plate’ is a custom-made force plate that only measures vertical force. The force transducer (Scaime AP200C3SH10eF, range 2000 N) is mounted in a 60 x 50 x 10 cm wooden box with a plywood plank 42 x 22 cm, thickness 1.8 cm, on top, flush with the surface of the box. Both surfaces are covered with a layer of closed-cell foam with a thickness of 0.6 cm. The force transducer was calibrated with known weights. Before each measuring session the force signal of the unloaded transducer is automatically set to zero, the force threshold level and the minimum time between stamps are input into the program. The force signal is preamplified and low-pass filtered with a cut-off of 50 Hz by an amplifier Scaime CPJ. Then it is sampled at 1000 Hz. An example signal is given in Fig 5. The momentum of the foot impact is determined by integrating the force signal starting from 10 ms before up to a time 100 ms after a trigger level of 2 N has been passed. In this way only the first short force peak is included in the measured momentum. In a number of experiments it was determined that this part of the force signal is directly related to the momentum of the foot, the later part of the force signal seems related to indirect forces from leg and trunk, and is much more variable.

#### STRIKE

**Fig 6:**
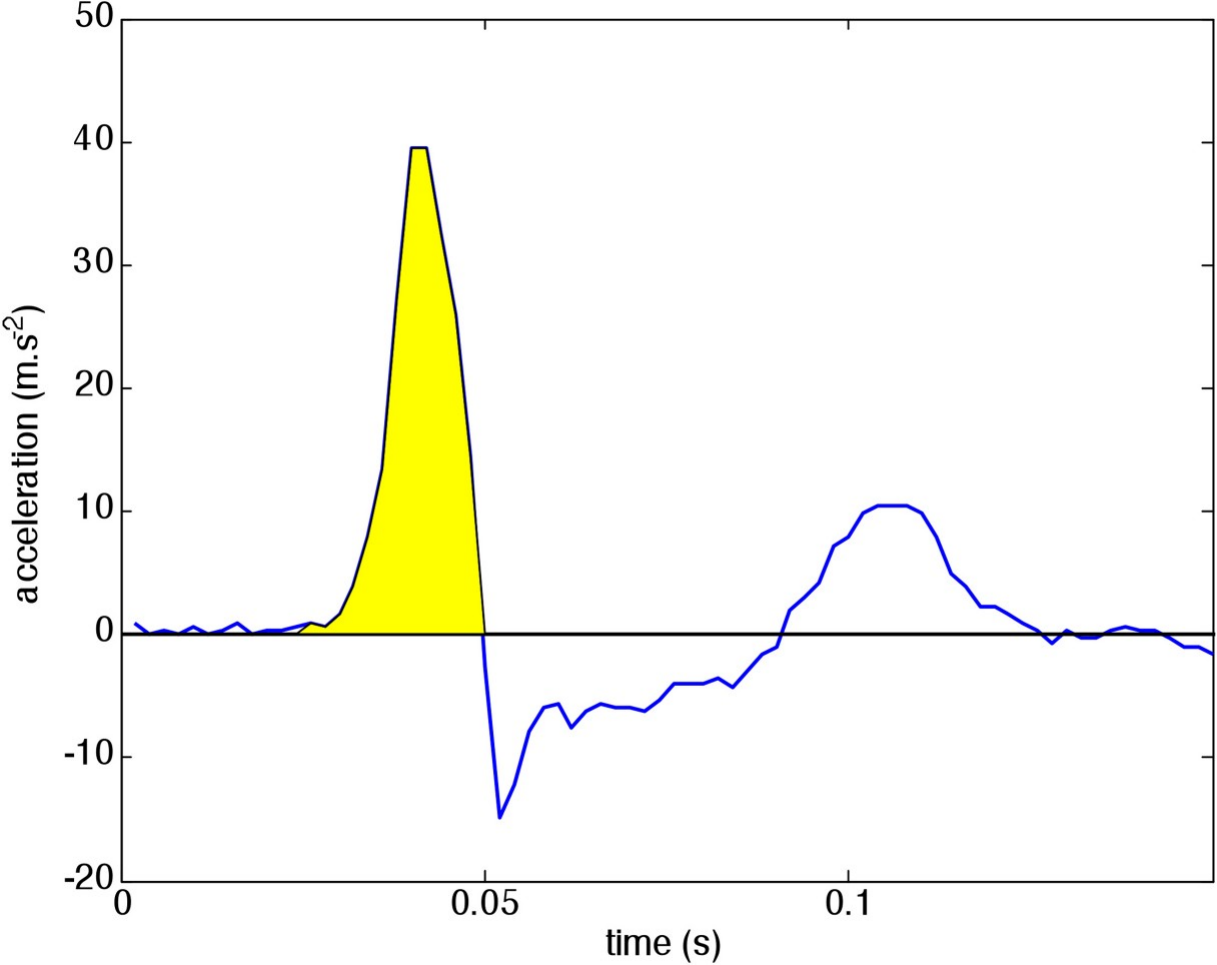
One of the two accelerometer signals in a strike on the punching bag. The impulses of the punch are equal to the shaded area under the curves times the mass of the punching bag $p_{x,y}=m_{bag}\int_{t_{1}}^{t_{2}}a_{x,y}dt$, with *t*_*1*_ 30 ms before the threshold (7 m·s^2^) is passed and *t*_*2*_ the time of the first zero crossing. The total impulse *p* is calculated as the vector sum of the two perpendicular components: $p=\sqrt{p_{x}^{2}+p_{y}^{2}}$. The accelerations measured after the first zero-crossing are due to vibrations and swinging of the bag after the strike.

The impact of the boxing strike is also expressed as a momentum. A dual-axis accelerometer ADXL278 (Analog Devices, range 50g) is mounted in a small box, with the sensitive axes horizontal. This box is put in the middle of a punching bag which is hung from the ceiling by a chain. The two perpendicular components of the acceleration are sampled at 500 Hz. The total momentum is calculated by integrating both acceleration signals (Fig 6), from 30 ms before a trigger level is passed up to the first zero crossing (the latest of the two components). The total impulse is calculated as the vector sum of both integrated accelerations (velocities) times the mass of the punching bag. In the present test the mass was 38 kg. Before the session this mass is input into the program, together with the trigger level, and the zero level of the accelerometers.

#### SHOUT

The sound is recorded by an USB desktop microphone (Logitech 980186–0914, -47 dBV/Pa, frequency range flat from 100 Hz– 11 kHz) via the PC sound card by means of dedicated LabVIEW programs. The participant is positioned 1.5 m from the microphone. Over periods of 0.1 s the sound signal is sampled at 22 kHz and the maximum sound level in dB over this period is calculated. It was verified that the recording was not distorted at the high sound levels as measured (up to 95 dB).

### Measures

The momentum of stamping and striking, and the amplitude of shouting are measured to quantify force production at different levels of executing the MSSS, that is 25%, 50%, 75%, 100% and back to 75%, 50% and 25% of maximum force. A series of numeric symbols represent momentum *p* as parameter for the Stamp and Strike force production, replaceable by amplitude *A* as parameter for loudness of the Shout: $p_{25↑};p_{50↑};p_{75↑};p_{100};p_{75↓};p_{50↓};p_{25↓}$. The arrows indicate the increasing (↑) and decreasing (↓) part of the force pyramid. Additionally, extra Shout parameters indicate shouting with short and long duration and the time span when shouting long: ${Db}_{short},{Db}_{long},T_{long}$. The amplitude of the Shout is directly related to sound-intensity measured in decibel on a logarithmic scale. The rule of thumb is that three decibels more means two times more sound-intensity. However, differences exist between actual values and perception of loudness: it takes a 10dB increase before the average listener hears ‘double the sound.’ (http://www.acousticsbydesign.com/acoustics-blog/perception-vs-reality.htm).

The $p_{SUM}$ represents the sum of all force levels per subtest and is used as an operationalization for the overall force produced. Linear and quadratic contrast scores (C↑, C↓, QC↑, QC↓) were calculated as operationalizations of force control increasing and decreasing. The use of these contrast scores is recommended by Davis [32] as a way to handle a sequence of measures from individuals and gain insight in the shape of the association between, for instance, dose and response, or the line representing treatment outcomes over time. The clustering of measures within persons is overcome by using an aggregate outcome representing the spacing and curve of the line over all measures. These aggregate scores make it possible to test whether treatment results increase with higher dosages or later time points (linear contrast) and whether there is an optimal level above which a higher dosage or longer treatment time has no or a diminishing effect (quadratic contrast). In our study, contrast scores give an indication of the steepness and shape of the slopes when increasing and decreasing stamping, striking or shouting force, showing the propensity to accelerate or hesitate when moving to the subsequent levels of force production. The amount by which single levels of force production differ from a straight line may be seen as the ‘error’ in force distribution, i.e. force control. Following Davis [32], we calculated the linear contrast scores by assigning coefficients of –3, –1, +1, and +3 to the force levels of the increasing half of the pyramid slope (indicated by the symbol *C*↑) respectively the decreasing half of the slope (*C*↓) and the quadratic contrast scores (*QC*↓ and *QC*↑) subsequently by assigning coefficients of –1, +1, +1, -1.

To assess the relationship between test performance and anger coping, the Self-Expression and Control Scale (SECS) was used to measure anger expression and control [33]. This questionnaire served as a frame of reference to explore the test validity of the MSSS. The SECS is a Dutch adaptation of the State Trait Anger Expression Inventory (STAXI) [34]. The STAXI is widely used to investigate the role of anger expression and anger control in somatic and mental health care. The SECS consists of 40 items divided into 4 subscales: Anger In (AI), Anger Out (AO), Control Anger In (CAI) and Control Anger Out (CAO). The AI subscale assesses efforts to hide anger (anger internalization), whereas the AO subscale assesses outwardly directed anger (anger externalization). The CAI and CAO subscales refer to the ability to modulate emotional and behavioural expression of anger (control over internalization and externalization). The SECS subscales have high levels of internal consistency and test-retest reliability [35]. AI was found to be correlated with cynicism, cynical distrust and indirect aggression; AO with aggressive responding and direct aggression [36].

### Statistical analyses

Not all data from the 104 participants could be used. Based on visual inspection of the irregular scores, scores ± 3 standard deviations from the mean were removed. One participant could not execute the subtest Stamp because the force plate failed to process the measuring data. The final data set contained 94 participants for Stamp, 98 for Strike and 95 for Shout.

Statistical analyses were performed in SPSS version 22. Without the outliers, the data of the parameters for all three subtests were normally distributed. Pearson’s correlations were calculated to test for possible confounders: the association between frequency of exercising and the stamping parameters, between exercising as well as boxing experience with striking, and the influence of trained vocal skills on shouting. Since weight and gender can be assumed to correlate with the outcomes of all three subtests, ANCOVA’s were executed to test for differences between men and women with weight controlled for.

An explorative search is used to detect patterns of performance. Pearson’s correlations between parameters within each subtest were calculated to describe the relationship between parameters, both within and between the increasing and decreasing parameters. Cronbach’s alpha over all parameters of each of the three subtests was calculated as an estimate of the internal consistency of that subtest. To investigate whether the three subtests are interrelated, Pearson’s correlations between the subtests on the different parameters were calculated, using partial correlations to control for gender and weight. In line with Cohen [37], we interpreted correlations between 0.10 and 0.30 as small, between 0.30 and 0.50 as medium and larger than 0.50 as indications of a strong association between outcomes.

The test-retest reliability of the MSSS was tested by the Intraclass Correlation Coefficient (ICC) for 22 participants that executed the test twice under similar laboratory conditions. A two samples independent t-test was done to check whether the mean sum scores on the extra test of the 22 participants deviated significantly from the scores of the 82 participants who were only tested once.

To test the construct validity of the MSSS, the correlations between the different parameters of the MSSS subtests and the four scales of the SECS were calculated, including sum scores, linear contrast scores, and quadratic contrast scores. As weight and gender were found to contribute to the variance of the MSSS subtests, partial correlations were used controlled for weight, and analyses were done separately for men and women. The SECS scores of participants were compared with those of the available SECS reference groups [33]. Further, a more robust comparison was made between two groups of participants respectively showing an internalizing versus an externalizing anger coping style. Those with higher AI scores than AO scores were referred to the internalizing group, and vice versa. Five participants (four female and one male) had equal scores on both subscales. They could not be included in one of the two groups and were left out of the analyses. Differences between both groups were calculated for men and women with ANCOVA, adjusted for weight. For these analyses we used listwise deletion of participants with missing values, resulting in smaller but comparable groups for the three subtests.

As we stated in the introduction, our study has an explorative character for there is too little consistent prior knowledge to conduct confirmative hypothesis testing. As a consequence, we refrained from presenting significance levels (p-values) for correlations where the outcomes on single force levels are concerned. Presenting significance levels for each of these correlations would possibly lead to faulty interpretations triggered by random findings due to multiple testing and the fact that in these cases the clustering of data within persons is ignored. We do, however, present significance levels for the aggregated (sum and contrast) scores. In these cases we follow the so-called neoFisherian approach [38] and interpret the p-value as a degree of evidence against the null- hypothesis that the difference is zero. To help interpretation of differences, confidence intervals are shown. This neoFisherian approach fits very well with the explorative nature of our inferences. Also visual inspection was used to asses correlations and differences.

## Results

### Participants characteristics

Table 1 presents demographic data and other characteristics of the participants, all students at university or other institutes of higher education. After testing for possible confounders no correlations were found between the frequency of exercising during the week, the mean scores on the various levels of the force pyramid or any other research parameters in both Stamp and Strike subtests. Boxing experience was not significantly correlated with any of the research parameters for striking. And, no significant correlation was found for trained vocal skills and any of the research parameters for shouting. Male and female participants differed on height and weight: men in our sample were taller and heavier.

**Table 1:** Participants characteristics (n = 104).^a^

	Men	Women	Difference between participating men and women^b^
	n (%)	n (%)	p
Participants	48 (46%)	56 (54%)	
Boxing experience	8 (17%)	6 (11%)	.43
Trained vocal skills	3 (6%)	5 (9%)	.61
	M (SD)	M (SD)	
Age (years)	20.88 (2.26)	20.80 (2.32)	.87
Height (m)	1.84 (.08)	1.73 (.06)	< .001
Weight (kg)	75.59 (10.14)	65.81 (8.25)	< .001
Body Mass Index	22.37 (2.03)	21.94 (2.24)	.29
Physical exercise (#/week)	2.79 (2.11)	2.36 (1.59)	.24

^a^ Since no outliers occurred simultaneously in all subtests, the total group characteristics have been included

^b^ Based on Fisher–Exact and independent t-test

Table 2 shows significantly higher scores on AO and CAO for male participants compared with the mean scores of a Dutch reference sample [33]. Female participants score lower on AO, and much higher on CAI and CAO compared with the population sample. No differences on the subscales of the SECS were found between scores of female and male participants in the study sample.

**Table 2:** SECS subscale scores of participants, comparison with reference groups and difference between men and women.

	Women (n = 56)			Men (n = 48)			Difference between Participating men and women
	Participants	Reference group^b^		Participants	Reference group^b^		
SECS^a^	M (SD)	M (SD)	p	M (SD)	M (SD)	p	p
AI	23.02 (5.70)	22.1 (7.0)	.23	22.17 (5.07)	23.1 (6.9)	.21	.43
AO	20.50 (5.28)	22.0 (5.9)	.04	21.92 (4.62)	20.2 (5.2)	.01	.15
CAI	28.79 (5.19)	24.9 (6.4)	< .001	28.29 (5.95)	27.3 (7.1)	.25	.65
CAO	30.32 (4.98)	25.6 (6.2)	< .001	31.02 (4.87)	29.5 (6.0)	.04	.47

^a^ Self-Expression and Control Scale; Anger In (AI; internalization), Anger Out (AO; externalization), Control Anger In (CAI), Control Anger Out (CAO).

^b^ As reported by Van Elderen et al, 1994 [33]

### Total scores and the influence of gender and weight

There was a positive correlation between weight and all seven parameters of the force pyramid for Stamp (0.43 < r > 0.49). Weight was also significantly positively correlated with all seven parameters of the force pyramid for Strike (0.47 < r > 0.55) and for Shout (0.40 < r > 0.47).

Table 3 shows the mean force production scores on the measured parameters, including the sum scores and the extra parameters for shouting with short and long duration and the time span when shouting long. For all subtests weight and gender explained a significant amount of variance in force production. On the Stamp subtest the linear contrast scores show that women displayed a stronger rate of increase and decrease in force production. On the Strike subtest, the linear contrast scores show that men have a stronger rate of force increase than women, with no gender differences in rate of force decrease. The Shout subtest shows no differences in linear contrast scores.

**Table 3:** MSSS Stamp, Strike, Shout subtests: mean force at different levels of force, mean sum and both linear and quadratic contrast scores, standard deviations and confidence intervals (CI = 95%) for men and women.^a^

**Stamp** (n = 94)	**Men** (n = 40)		**Women** (n = 54)	
	Mean (SD)	CI	Mean (SD)	CI
$p_{25↑}$	17.7 (5.71)	15.2–20.1	12.1 (4.66)	10.4–13.8 ^b.c^
$p_{50↑}$	20.6 (5.63)	18.2–23.0	15.1 (5.45)	13.1–17.1 ^b,c^
$p_{75↑}$	22.4 (5.85)	19.9–24.9	17.5 (6.08)	15.3–19.7 ^b,c^
$p_{100}$	24.8 (5.83)	22.3–27.3	22.0 (6.11)	19.8–24.2 ^b,c^
$p_{75↓}$	22.4 (6.29)	19.7–25.1	17.3 (6.21)	15.1–19.6 ^b,c^
$p_{50↓}$	20.9 (6.37)	18.2–23.7	15.3 (5.82)	13.2–17.4 ^b,c^
$p_{25↓}$	18.5 (6.32)	15.8–21.2	12.6 (5.51)	10.6–14.6 ^b,c^
$p_{SUM}$	**147.3 (39.4)**	**134.7–159.9**	**111.9 (38.2)**	**101.5–122.3** ^b,c^
***C↑***	**23.2 (17.9)**	**17.5–28.9**	**32.3 (14.0)**	**28.4 − 36.1** ^b,c^
***C*↓**	**20.2 (17.3)**	**14.7–25.7**	**30.2 (12.3)**	**26.8–33.5** ^b,c^
***QC*↑**	**-0.44 (3.38)**	**-1.52–0.64**	**1.45 (3.30)**	**0.55–2.35**
***QC*↓**	**0.04 (3.05)**	**-0.93–1.02**	**2.03 (2.89)**	**1.24–2.81**^b^
**Strike** (n = 98)	**Men** (n = 44)		**Women** (n = 54)	
	Mean (SD)	CI	Mean (SD)	CI
$p_{25↑}$	11.5 (3.97)	9.87–13.1	6.58 (3.00)	5.49–7.67 ^b,c^
$p_{50↑}$	15.1 (4.91)	13.1–17.1	8.51 (3.20)	7.34–9.67 ^b,c^
$p_{75↑}$	18.3 (6.82)	15.5–21.1	10.8 (3.43)	9.56–12.1 ^b,c^
$p_{100}$	21.9 (6.65)	19.2–24.6	14.6 (3.98)	13.2–16.1 ^b,c^
$p_{75↓}$	17.0 (5.21)	14.8–19.1	10.4 (3.25)	9.22–11.6 ^b,c^
$p_{50↓}$	15.5 (5.53)	13.3–17.8	8.65 (2.99)	7.57–9.74 ^b,c^
$p_{25↓}$	12.9 (4.65)	11.1–14.8	7.04 (2.67)	6.07–8.01 ^b,c^
$p_{SUM}$	**112.3 (34.0)**	**101.9–122.6**	**66.6 (20.2)**	**61.1–72.1** ^b,c^
***C*↑**	**34.5 (17.4)**	**29.2–39.7**	**28.4 (15.6)**	**23.3–29.4** ^b,c^
***C*↓**	**26.4 (11.1)**	**23.6–33.1**	**24.5 (10.8)**	**21.5–27.4**
***QC*↑**	**-0.39 (5.50)**	**-1.71–1.63**	**1.88 (3.01)**	**1.06–2.71**
***QC*↓**	**2.38 (4.35)**	**1.05–3.70**	**2.59 (2.89)**	**1.80–3.37**
**Shout** (n = 95)	**Men** (n = 46)		**Women** (n = 49)	
	Mean (SD)	CI	Mean (SD)	CI
$A_{25↑}$	74.8 (5.53)	72.6–77.0	67.6 (6.35)	65.2–70.1 ^b,c^
$A_{50↑}$	79.7 (5.40)	77.5–81.8	72.1 (6.33)	69.6–74.5 ^b,c^
$A_{75↑}$	83.6 (5.48)	81.4–85.8	76.1 (6.78)	73.5–78.7 ^b,c^
$A_{100}$	88.8 (6.00)	86.4–91.1	82.0 (6.81)	79.4–84.7 ^b,c^
$A_{75↓}$	82.6 (6.48)	80.0–85.1	74.1 (7.60)	71.2–77.0 ^b,c^
$A_{50↓}$	78.8 (6.27)	76.3–81.3	70.5 (7.26)	67.7–73.3 ^b,c^
$A_{25↓}$	73.9 (7.06)	71.1–76.7	65.7 (7.72)	62.7–68.6 ^b,c^
$A_{SUM}$	**562.1 (39.6)**	**550.3–573.9**	**508.2 (44.8)**	**495.3–521.1** ^b,c^
***C↑***	**45.9 (12.1)**	**42,3 − 49,5**	**47.3 (18.4)**	**42.0–52.5**
***C↓***	**48.3 (15.4)**	**43,7 − 52,9**	**52.6 (21.4)**	**46.5–58.7**
***QC*↑**	**0.24 (3.40)**	**-0.77–1.25**	**1.48 (4.05)**	**0.32–2.65**
***QC*↓**	**48.3 (15.5)**	**43.7–52.9**	**52.7 (21.4)**	**46.5–58.8**
${Db}_{short}$	74.6 (5.70)	72.8 − 76.2	67.9 (7.38)	65.8–69.9 ^b,c^
${Db}_{long}$	65.7 (8.27)	63.4–67.9	60.1 (8.42)	57.8–62.4 ^b,c^
$T_{long}$	19.3 (8.45)	16.8–21.8	14.4 (5.45)	13.0–15.9 ^b,c^
$A_{SUM-extra}$	**721 (49)**	**707.0–736.3**	**650 (59)**	**633.5–667.6** ^b,c^

^a^ Momentum p for the subtests Stamp and Strike is given in kg·m/s, amplitude A of the Shout in dB, T_long_ in seconds; see section Measures: force parameters

^b,c^ Differences between men and women explained by gender alone^b^ and controlled for weight^c^ (p<0.01)

For the quadratic contrast scores no differences were found for men and women with the exception of the stamp subtest decreasing: women scored higher than men indicating a more pronounced curve.

Both weight and gender were taken as confounder in the following calculations and tests.

**Fig 7:**
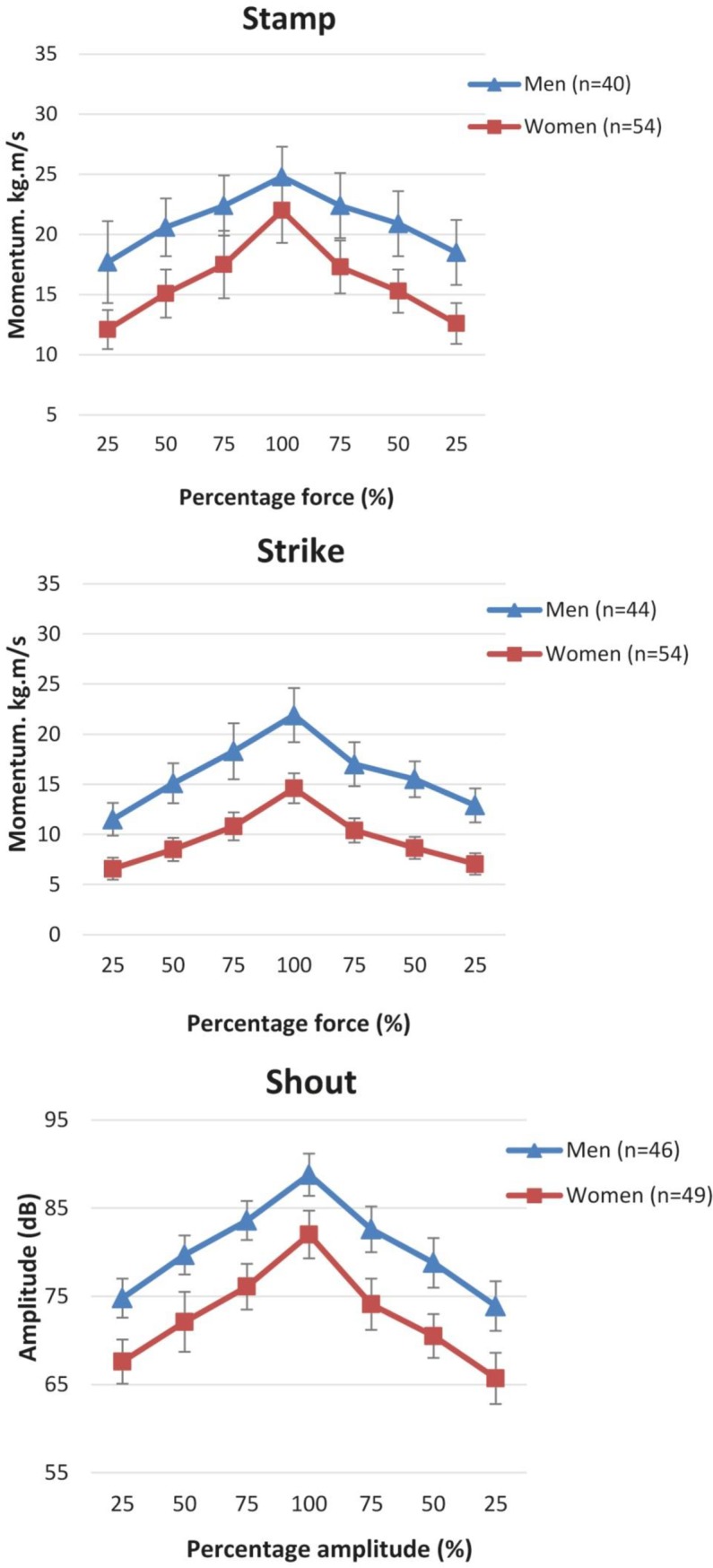
Force pyramids (mean, CI 95%) of each subtest of the MSSS for men and women.

Fig 7 presents the force pyramids of each subtest performed by men and women. Visual inspection shows that both men and women were able to perform a pyramid-like performance of force production in all three subtests. For women performing the Stamp subtest it seems the step sizes around 100% force were relatively large.

### Internal consistency

To measure Cronbach’s alpha all parameters of each subtest were used as a scale. For the Stamp subtest α = 0.98, for the Strike subtest α = 0.97, for the Shout subtest α = 0.98, indicating an excellent internal consistency.

### Test-retest reliability

The ICC’s between test and retest of 22 participants were high for all parameters, indicating a strong test-retest resemblance in force production on each of the levels (Table 4). The ICC is also high for the linear contrast scores. For the quadratic contrast scores the ICC is medium within the strike subtest and low within the Shout subtest increasing. There were no significant differences between the retest mean sum scores of these 22 participants and the mean sum scores of the other 82 participants (respectively: mean Stamp sum score 132 (SD = 62) and 126 (SD = 41), t = 0.42, df = 24.79, p = 0.68; mean Strike sum score 88 (SD = 39) and 85 (SD = 33), t = 0.33, df = 97, p = 0.74; mean Shout sum score 524 (SD = 59) and 532 (SD = 47), t = -0.61, df = 94, p = 0.54).

**Table 4:** Test-retest reliability for the parameters of the MSSS, assessed by the Intraclass Correlation Coefficient (ICC).^a^

	**Stamp**	**Strike**	**Shout**
	ICC	ICC	ICC
$p_{25↑}$^b^	.82	.49	.84
$p_{50↑}$	.91	.72	.90
$p_{75↑}$	.92	.82	.88
$p_{100}$	.85	.72	.93
$p_{75↓}$	.93	.82	.91
$p_{50↓}$	.92	.79	.94
$p_{25↓}$	.83	.58	.82
$p_{SUM}$	**.94**	**.89**	**.91**
***C↑***	**.81**	**.69**	**.89**
***C↓***	**.79**	**.66**	**.88**
***QC↑***	**.54**	**.44**	**.26**
***QC↓***	**.64**	**.47**	**.83**
${Db}_{short}$			.80
${Db}_{long}$			.83
$T_{long}$			.85
$A_{SUM-extra}$			**.91**

^a^ Momentum *p* in kg·m/s for the subtests Stamp and Strike can be replaced by amplitude *A* in dB for the Shout subtest.

^b^ See section Measures: force parameters

### Intra-test correlations

Tables 5–7 show that the force parameters were highly inter-correlated. Obviously, high correlations existed between the parameters that succeed one after another within the increasing and decreasing part of the sequence. These correlations were somewhat less strong between 100% and the bottom force levels. High correlations also existed between the corresponding parameters of the increasing and decreasing part of the pyramid, for example between $p_{50↑}$ and $p_{50↓}$, especially very high for the Stamp test. The correlations confirm that most participants were able to increase force gradually to 100% and decrease force gradually to 25% again following a pyramid like sequence (Fig 7). For the Shout subtest, the extra correlations between the sequential parameters and shouting with short duration were high, as was the correlation between shouting with short and long duration. The correlations between the time span when shouting long and most other parameters were moderate.

**Table 5:** Intra-test correlations (Pearsons r) Stamp (n = 94), controlled for gender and weight.

Stamp^a^	$p_{25↑}$	$p_{50↑}$	$p_{75↑}$	$p_{100}$	$p_{75↓}$	$p_{50↓}$	$p_{25↓}$
$p_{25↑}$	-	.91	.81	.59	.79	.88	.94
$p_{50↑}$		-	.93	.75	.91	.95	.93
$p_{75↑}$			-	.87	.96	.93	.86
$p_{100}$				-	.89	.79	.68
$p_{75↓}$					-	.95	.87
$p_{50↓}$						-	.96
$p_{25↓}$							-

^a^ See section Measures: force parameters

**Table 6:** Intra-test correlations (Pearsons r) strike (n = 98), controlled for gender and weight.

Strike^a^	$p_{25↑}$	$p_{50↑}$	$p_{75↑}$	$p_{100}$	$p_{75↓}$	$p_{50↓}$	$p_{25↓}$
$p_{25↑}$	-	.81	.71	.56	.74	.76	.81
$p_{50↑}$		-	.83	.67	.81	.78	.75
$p_{75↑}$			-	.77	.81	.79	.66
$p_{100}$				-	.79	.71	.60
$p_{75↓}$					-	.84	.75
$p_{50↓}$						-	.84
$p_{25↓}$							-

^a^ See section Measures: force parameters

**Table 7:** Intra-test correlations (Pearsons r) shout (n = 95), controlled for gender and weight.

Shout^a^	$A_{25↑}$	$A_{50↑}$	$A_{75↑}$	$A_{100}$	$A_{75↓}$	$A_{50↓}$	$A_{25↓}$	${Db}_{short}$	${Db}_{long}$	$T_{long}$
$A_{25↑}$	-	.88	.81	.66	.77	.80	.80	.62	.41	.28
$A_{50↑}$		-	.94	.82	.88	.87	.79	.60	.43	.28
$A_{75↑}$			-	.87	.89	.86	.74	.65	.49	.34
$A_{100}$				-	.86	.75	.64	.53	.45	.31
$A_{75↓}$					-	.91	.81	.58	.46	.45
$A_{50↓}$						-	.89	.61	.45	.33
$A_{25↓}$							-	.58	.45	.35
${Db}_{short}$								-	.62	.29
${Db}_{long}$									-	.06
$T_{long}$										-

^a^ See section Measures: force parameters

Intra-test correlations were also calculated for the aggregated scores: for the (linear and quadratic) contrast scores of the two slopes within each pyramid and between these and the sum score for each pyramid, using p<0.01 as level of significance (no table). Regarding the linear contrast scores, the intra correlations within the same subtest between C↑ and C↓ were significantly high: for Stamp 0.91, for Strike 0.85 and for Shout 0.73. Regarding the quadratic contrast scores, the intra correlations within the same subtest between QC↑ and QC↓ were significantly high for Stamp and for Strike, respectively 0.64 and 0.61, but for Shout no significant intra correlation between QC↑ and QC↓ was found.

Sum scores and linear contrast scores within the Stamp and Shout subtests were not correlated, but for the Strike subtest a significant small respectively medium correlation was found between sum scores and linear contrast scores: 0.27 (↓) respectively 0.34(↑).

Significant medium correlations were found between sum scores and quadratic contrast scores for the Stamp subtest: -0.43 (↑) and -0.50(↓). No other correlations were found for Strike or Shout.

### Inter-test correlations

The force parameters of each subtest and their sum scores were medium correlated between the tests (Table 8). No correlations between subtests were found for the linear and quadratic contrast scores.

**Table 8:** Inter-test correlations (Pearsons r) for all subtests, controlled for gender and weight using partial correlations.^a^

	Stamp & Strike (N = 90)	Stamp & Shout (N = 87)	Strike & Shout (N = 90)
$p_{25↑}$^b^	.48	.36	.27
$p_{50↑}$	.41	.34	.28
$p_{75↑}$	.33	.36	.30
$p_{100}$	.36	.26	.35
$p_{75↓}$	.42	.40	.39
$p_{50↓}$	.42	.31	.34
$p_{25↓}$	49	.33	.34
$p_{SUM}$	**.47**	**.37**	**.27**
**C↑**	**. 11**	**.16**	**.22**
**C↓**	**. 19**	**.10**	**.13**
**QC↑**	**.13**	**.06**	**-.17**
**QC↓**	**.27**	**-.08**	**-.01**

^a^ Momentum *p* can be substituted by amplitude *A*.

^b^ See section Measures: force parameters

### Associations between levels of force production and anger coping

The Stamp subtest (Table 9) performed by women (n = 54) reveals a pattern of low negative correlations between the force levels and an AI coping style. The significant medium correlation between the sum score of force parameters and AI indicates that less force was produced by those participants who have higher scores of AI on the SECS. Stamping force produced by women shows no

correlation patterns with AO, nor with CAI and CAO. In the Stamp subtest performed by men no clear correlation patterns can be seen with the SECS subscales.

**Table 9:** Association (Pearson’s *r*) between the Stamp and SECS^a^ outcomes, apart for men and women and controlled for weight (p = m kg/sec).

Stamp	Women (n = 54)				Men (n = 40)			
	AI	AO	CAI	CAO	AI	AO	CAI	CAO
$p_{25↑}$^b^	-.29	.16	-.23	-.08	-.03	.03	-.17	-.29
$p_{50↑}$	-.23	.05	-.17	-.05	-.05	.02	-.20	-.19
$p_{75↑}$	-.24	.09	-.15	-.02	-.08	.05	-.28	-.22
$p_{100}$	-.17	.15	-.08	-.08	-.08	.05	-.18	-.04
$p_{75↓}$	-.29	.09	-.12	-.04	-.10	.06	-.25	-.18
$p_{50↓}$	-.28	.07	-.12	-.01	.02	.08	-.28	-.30
$p_{25↓}$	-.27	.10	-.16	-.003	.02	.06	-.23	-.28
$p_{SUM}$	-.30*	**.08**	**-.13**	**-.10**	**-.03**	**.02**	**-.21**	**-.27**
**C↑**	**.10**	**-.05**	**.19**	**.07**	**-.05**	**.11**	**-.21**	**.18**
**C↓**	**.12**	**.02**	**.14**	**-.04**	**-.09**	**.05**	**-.07**	**.28**
**QC↑**	**.15**	**.16**	**.05**	**-.09**	**.03**	**-.01**	**.12**	**.15**
**QC↓**	.48**	**.01**	**-.03**	**-.03**	**.00**	**-.14**	**.28**	**.37**

^a^ Self-Expression and Control Scale; Anger In (AI; internalization), Anger Out (AO; externalization), Control Anger In (CAI), Control Anger Out (CAO).

^b^ See section Measures: force parameters.

* p<0.05

** p<0.01 (the significance of correlations was tested for the sum and contrast scores only)

The Strike subtest (Table 10) performed by women as well as men showed no correlation patterns with the subscales of the SECS.

**Table 10:** Association (Pearson’s *r*) between the Strike and SECS^a^ outcomes, apart for men and women and controlled for weight (p = m kg/sec).

Strike	Women (n = 54)				Men (n = 44)			
	AI	AO	CAI	CAO	AI	AO	CAI	CAO
$p_{25↑}$^b^	-.12	-.01	.07	.05	-.03	-.13	.02	.11
$p_{50↑}$	-.16	.06	.08	.002	.04	-.17	-.03	.19
$p_{75↑}$	-.09	.04	.23	.05	-.07	-.20	-.02	.25
$p_{100}$	-.14	.01	.17	-.02	-.22	.01	-.05	.08
$p_{75↓}$	-.19	.06	.03	-.05	-.17	-.15	.07	.24
$p_{50↓}$	-.18	.09	.08	-.01	-.04	-.10	.09	.13
$p_{25↓}$	-.19	.08	.00	.03	-.16	-.01	-.11	-.02
$p_{SUM}$	**-.19**	**.08**	**.10**	**-.01**	**-.13**	**-.18**	**.04**	**.13**
**C↑**	**.04**	**.01**	**.18**	**-.08**	**-.30**	**.16**	**-.21**	**.03**
**C↓**	**.04**	**-.02**	**.18**	**-.09**	**-.16**	**.07**	**-.13**	**.06**
**QC↑**	**.08**	**-.03**	**-.04**	**-.01**	**-.07**	.40*	**-.27**	-.46**
**QC↓**	**.13**	**-.01**	**.14**	**.06**	**-.25**	**.33**	-.45**	-.35*

^a^ Self-Expression and Control Scale; Anger In (AI; internalization), Anger Out (AO; externalization), Control Anger In (CAI), Control Anger Out (CAO).

^b^ See section Measures: force parameters.

* p<0.05

** p<0.01 (the significance of correlations was tested for the sum and contrast scores only)

The Shout subtest (Table 11) performed by women showed the most and strongest correlation patterns. For women, negative medium correlations were found between AI and all force levels, including the extra parameters for shouting with short and long duration and the time span when shouting long. The sum scores of the force parameters and the extra shout parameters show a significant medium correlation with AI. The negative direction indicates that women higher in AI tend to use less force. For women, medium positive correlations were found between AO and all force levels, including the extra Shout parameters. The sum scores show significant medium correlations in a positive direction, indicating that those scoring high on AO produced more volume. For women, negative medium correlation patterns can be seen between the control scales (CAI and CAO) and the amplitude of the Shout: the more women control their voice, the lower the force production, which is confirmed by significant correlations for the sum scores of increasing and decreasing force parameters, as well as for the sum scores of the extra Shout parameters. The Shout subtest performed by men shows a negative medium correlation pattern between force levels and AI, as confirmed by a significant correlation for the sum score, though somewhat less strong compared with women (Table 11).

**Table 11:** Association (Pearson’s *r*) between the Shout and SECS^a^ outcomes, apart for men and women and controlled for weight (A = dB).

Shout	Women (n = 49)				Men (n = 46)			
	AI	AO	CAI	CAO	AI	AO	CAI	CAO
$A_{25↑}$^b^	-.29	.40	-.40	-.36	-.20	-.13	-.03	-.24
$A_{50↑}$	-.37	.41	-.36	-.39	-.36	-.01	-.14	-.23
$A_{75↑}$	-.43	.41	-.29	-.37	-.31	-.14	.01	-.12
$A_{100}$	-.42	.23	-.19	-.25	-.24	-.13	-.03	-.01
$A_{75↓}$	-.41	.31	-.24	-.25	-.30	-.15	.004	.01
$A_{50↓}$	-.39	.45	-.37	-.36	-.32	-.19	-.04	-.05
$A_{25↓}$	-.40	.45	-.35	-.35	-.29	-.08	-.07	-.06
$A_{SUM}$	-.46**	.38**	-.37**	-.41**	-.30*	**-.13**	**-.04**	**-.12**
**C↑**	**-.21**	**-.15**	**.19**	**.06**	**-.02**	**-.04**	**-.02**	**.15**
**C↓**	**-.03**	**-.28**	**.17**	**.14**	**.07**	**-.09**	**.01**	**.17**
**QC↑**	**.09**	**-.28**	**.06**	**.17**	**.21**	**-.19**	**.06**	**.11**
**QC↓**	**-.03**	**-.28**	**.17**	**.14**	**.07**	**-.09**	**.01**	**.17**
${Db}_{short}$	-.45	.33	-.30	-.28	.18	-.11	.05	-.29
${Db}_{long}$	-.38	.10	-.05	-.14	.28	-.08	-.10	-.13
$T_{long}$	-.34	.21	-.17	-.30	-.25	-.20	.04	-.03
$A_{SUM-extra}$	-.50**	.36*	-.34*	-.40**	**-.21**	**-.17**	**-.03**	**-.16**

^a^ Self-Expression and Control Scale; Anger In (AI; internalization), Anger Out (AO; externalization), Control Anger In (CAI), Control Anger Out (CAO).

^b^ See section Measures: force parameters.

* p<0.05

** p<0.01 (the significance of correlations was tested for the sum and contrast scores only)

The linear contrast scores for each subtest show no significant correlation with de SECS subscales. The quadratic contrast scores show a significant positive correlation on the Stamp subtest with the AI subscale for women decreasing their force production. On the Strike subtest these quadratic contrast scores correlate negatively with the CAI subscale for men decreasing their force and with the CAO subscale for men increasing their force. On the Shout subtest no correlations were found between quadratic contrast scores and SECS subscales.

### Differences between internalizing and externalizing women and men

**Fig 8:**
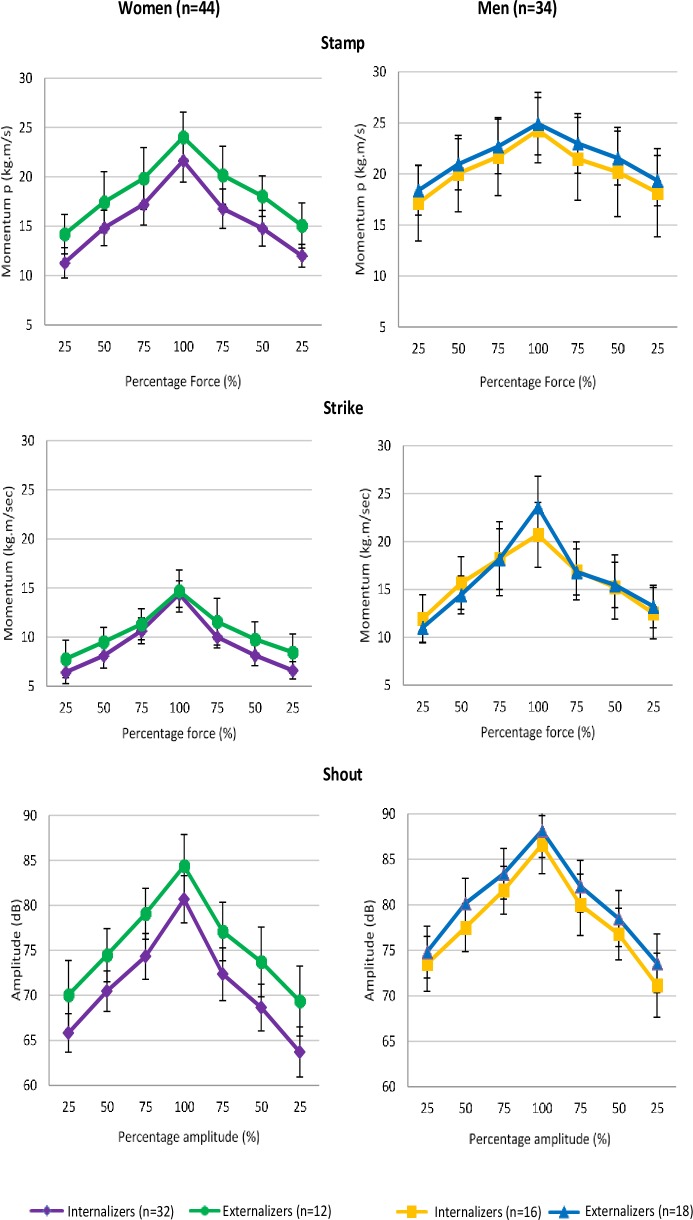
Force pyramids: differences (mean and CI 95%) in force production between internalizing and externalizing men (n = 34, defined respectively as scoring higher on AI than AO or vice versa) and women (n = 44) on the subtests of the MSSS.

Visual inspection of Fig 8 indicates that externalizing participants used more force than internalizing participants, particularly confirmed in the Shout subtest performed by women. In the Strike subtest it is only at the 100% force level that externalizing men produced more effort than internalizing men. Whereas women scored overall lower on force production, externalizing women used equal force as men, when stamping at 100% force level.

The outcomes on the extra parameters of the Shout subtest − shouting with short and long duration and the time span when shouting long–are presented in Table 12. The women showed a consistent trend: those with an externalizing anger coping style reached higher amplitudes and a longer time span.

**Table 12:** Differences (mean and CI 95%) between internalizing and externalizing women and men on the extra parameters of the Shout subtest: shouting with short and long duration and the time span when shouting long.

Shout extra^a^	Women (n = 44)		Men (n = 34)	
	Internalizing^b^ (n = 32)	Externalizing^b^ (n = 12)	Internalizing^b^ (n = 16)	Externalizing^c^ (n = 18)
	M (CI 95%)	M (CI 95%)	M (CI 95%)	M (CI 95%)
${Db}_{short}$	66.00 (63.78–68.22)	72.47 (66.97–77.96)	73.81 (70.84–76.78)	74.20 (70.89–77.51)
${Db}_{long}$	59.11 (55.99–62.22)	62.67 (58.07–67.28)	67.01 (62.82–71.19)	64.05 (58.82–69.27)
$T_{long}$	13.56 (11.70–15.42)	16.73 (12.73–20.74)	17.75 (13.30–22.20)	18.42 (13.64–23.20)

^a^ See section Measures: force parameters.

^b^ Internalizing participants, scoring higher on AI than on AO.

^c^ Externalizing participants, scoring higher on AO than on AI.

Differences in mean sum scores (Table 13) confirm the trend that the externalizing group produced more force than the internalizing group. In women, mean sum scores in the Shout subtest differed significantly between the internalizing and the externalizing group.

No significant differences were found for the internalizing and externalizing group on linear and quadratic contrast scores (C↑ and C↓; QC↑ and QC↓) with two exceptions for C↑ in the Strike subtest and QC↓: externalizing men showed a stronger rate of force increase when striking the punch bag than internalizing men and, when decreasing, a line less resembling the quadratic curve.

**Table 13:** Total pattern of differences between internalizing and externalizing participants performing the MSSS, represented by the sum, linear contrast and quadratic contrast scores of force production, separately for women and men (*p* = m.kg/sec and *A* = dB).

Sum and contrast scores^a^	Women (n = 44)		Men (n = 34)	
	Internalizing^b^ (n = 32)	Externalizing^c^ (n = 12)	Internalizing^b^ (n = 16)	Externalizing^c^ (n = 18)
	M (CI 95%)	M (CI 95%)	M (CI 95%)	M (CI 95%)
Stamp $p_{SUM}$	108.6 (95.4–121.6)	128.8 (108.4–149.1)	143.0 (116.5–169.5)	150.8 (134.0–167.7)
C↑	33.4 (28.1–38.7)	31.9 (24.6–39.1)	23.8 (15.9–30.1)	21.3 (10.1–32.5)
C↓	30.9 (26.4–35.3)	29.0 (21.2–36.6)	24.6 (10.9–28.4)	18.2 (8.82–27.5)
**QC↑**	.89 (-.27–2.05)	.95 (.71–2.60)	-.31 (-2.23–1.62)	-.33 (-2.08–1.42)
**QC↓**	2.08 (1.00–3.06)	.89 (-.21–1.99)	.78 (-1.09–2.64)	-.28 (-1.54-.98)
Strike $p_{SUM}$	64.34 (56.8–71.7)	73.12 (61.8–84.3)	111.2 (91.1–131.4)	112.7 (98.2–127.2)
C↑	26.3 (23.2–29.6)	22.5 (14.4–30.6)	**29.9 (22.3–35.4)**	39.9 (31.7–51.4)*
C↓	25.2 (21.5–28.8)	20.5 (13.8–27.0)	25.2 (18.9–33.5)	31.1 (24.1–41.0)
**QC↑**	2.06 (.94–3.18)	1.66 (-.04–3.34)	-1.24 (-3.57–1.10)	2.01 (-.59–4.61)
**QC↓**	2.84 (1.78–3.09)	1.78 (-.13–3.69)	**1.03 (-.95–3.00)**	4.53 (2.20–6.85)*
Shout $A_{SUM}$	**496.0 (479.6–512.3)**	528.1 (506.7–549.6)*	547.2 (527.6–566.7)	560.7 (541.3–580.0)
C↑	48.4 (41.4–55.5)	47.6 (35.7–59.5)	49.9 (3.05–48.8)	45.9 (38.2–48.5)
C↓	54.6 (46.5–62.8)	48.4 (35.6–61.2)	48.2 (40.2–58.9)	46.7 (40.0–54.6)
**QC↑**	1.72 (.17–3.27)	.87 (-1.16–2.91)	1.03 (-.53–2.58)	-.61 (-2.73–1.51)
**QC↓**	54.6 (46.5–62.8)	48.4 (35.7–61.2)	49.6 (40.2–58.9)	47.3 (40.0–54.6)
$A_{SUM-extra}$	**634.7 (613.2–656.2)**	680.0 (650.1–709.9)*	705.8 (679.7–731.8)	717.4 (693.1–741.6)

^a^ See section Measures: force parameters.

^b^ Internalizing participants, scoring higher on AI than on AO.

^c^ Externalizing participants, scoring higher on AO than on AI.

* p<0.05 controlled for weight.

## Discussion

This explorative study investigated the internal structure, reliability, and validity of the Method of Stamp Strike Shout (MSSS), a new performance-based measuring instrument meant to assess anger expression and control. It was tested in a sample of 104 Dutch students. The performance of the MSSS was quantified by measuring mean force levels with increasing and decreasing levels of force production that follow a pyramid-like sequence. To test the validity of the MSSS, we used an ‘emotion follows action’ design, that is, exploring whether and how physical force production itself predicts levels of reported anger expression, anger inhibition and anger control, without offering anger provoking stimuli prior to the performance. Firstly, force parameters of the MSSS were correlated with the scores on the anger coping scale (SECS). Secondly, a more robust comparison was made between two groups of participants showing an anger internalizing (AI) versus an anger externalizing (AE) coping style.

Visual inspection of the tables and figures showed patterns that tell us how the pyramids are shaped, the steepness of the slopes and their symmetry. In addition, contrast scores were calculated serving as an indicator for rates of increase or decrease and the propensity to accelerate or hesitate in moving to the subsequent levels of force production.

### Internal consistency and reliability of the MSSS

The MSSS showed an excellent internal consistency of each subtest. Further, the test-retest reliability of the MSSS pointed towards a high degree of precision and reproducibility of the routine, with the test-retest resemblance in the bottom force levels of the Strike test the least high.

A period of five months between test and retest was chosen in order to reduce remembrance of the first performance. This is even more relevant in case the MSSS should elicit an emotional experience that would have a memory-enhancing effect [39]. Over such a long period there is a greater risk of personal changes that could affect the scores. In this non-clinical sample, the force production presented on the MSSS at different levels, however, seems to reflect relatively stable personal characteristics of the participants. However, the two contrast scores calculated to indicate the rate and distribution of force over all levels were shown to be more sensitive to change with low test-retest reliability.

Prior to the performance of the MSSS no familiarization sessions were carried out. In this stadium of testing the aim was to assess spontaneous interaction of force production and emotion, without interference of cognitive learning. Furthermore, little research has investigated the need of such trials to establish high degrees of test-retest reliability in force characteristics. For comparison, in a study on force measures with physically active men similar high degrees of test-retest reliability were achieved without the need for familiarization sessions [40].

### Within and between subtests correlations

In performing the MSSS, the Stamp test showed the highest *within subtest* correlations for the different force levels, followed by the Shout test. Striking the boxing bag may have been the most vulnerable for irregularities with less high correlations between different levels on both side of the pyramid. However, where in the Stamp and Strike subtest rates of increase and decrease of force production were associated, in the Shout subtest the two slopes were shown to be differently curved. For all subtests the correlations between the 100% force level and some of the other parameters was somewhat lower. The maximum force level seems to be somewhat independent on the steps to get there. The same holds true for the total sum of force production, which was found to be mostly independent of rate of increase / decrease and curve, except on the Strike subtest for rate of increase and the Stamp subtest for the curve.

The correlations found *between* the three subtests all remained under 0.50 (as was indicated as a strong correlation), with no indication found for associations between rate of increase / decrease of force production and curve. So where there is a common factor in the different subtests, they measured force production in a different way and thus may complement each other. Particularly the Shout subtest seemed to measure a different aspect.

### Associations between force production and anger coping

The Shout subtest was the most sensitive indicator for anger coping style in the student sample, showing moderate negative correlations with anger internalization, for women as well as men. A strong negative correlation with anger internalization was found when all parameters for shouting were taken together including measures for the amplitude of short and long shouts and time span for these long shouts. Women showed a more complete pattern: apart from the associations with anger internalization, they displayed a moderate positive relation between test performance on the Shout subtest with anger externalization, and moderate negative associations with anger control (CAI and CAO). In women performing the Stamp subtest, inhibition of force production correlated moderately with an internalizing style of anger coping (AI). In the Stamp subtest performed by men there was only a small negative association with the CAI and CAO scales. In the Strike subtest for women nor for men correlation patterns could be detected for force production at each of the specific levels.

More robust differences could be recognized when dividing the sample in an internalizing versus externalizing group (AI versus AO), for women and men separately. Visual inspection confirms the findings for women in the first analysis, for the parameters of the pyramid as well as the extra Shout parameters. Externalizing men showed extra force production at the 100% level of the Strike. Also the contrast score signifying force control were found to differentiate between internalising and externalising men. All in all, these explorative findings suggest that without having received anger evoking stimuli prior to the test, internalizing women as well as men used less force than externalizing participants on especially the Shout and, although less distinguished, the Stamp subtest of the MSSS. In the Strike subtest not the force expression at specific levels as much as the maximum, rate and distribution of force are of importance.

For now, there is no solid explanation for the supremacy of the Shout test when distinguishing between anger coping styles, especially where women are concerned. Perhaps voice expression is the skill with the most relational impact, more regulated by shame or negative appraisal, even more so in case anger was experienced. This may apply to the produced as well as perceived sound intensity.

The women in our student sample scored higher on the anger control scales than men, and also higher than the women of the reference population. Moreover, women scored lower on AO and men scored higher on AO compared with the norm. Also, in our sample twice as many women could be typified as internalizing compared with those externalizing, whereas the number of internalizing and externalizing men was practically the same. Smits and Kuppens [41] found more inhibition of physical aggression in women as compared with men. They argue that women more than men consider aggressive behaviour to be socially unacceptable and a reason to be concerned about negative self-evaluation and negative effects on others. In mental health care it can be recognized that many women still learn to silence their anger, deny it entirely, or vent it in another way. We found excessive anger internalization in women with eating disorders [20,21]. Although this may explain the gender differences also found in our present study, it does not account for the high incidence of anger internalization in our female student sample.

### Theory, research and clinical practice

To our knowledge, the combination of anger coping styles and force production in voice expression and in directional movements of arms and legs has not been previously investigated. As such, the MSSS is new in this field of research. Also, the test design in the present study differs from other studies. Most studies applied an ‘action follows emotion’ design by measuring force production after offering anger versus neutral stimuli. Starting point of the present study was to explore the anger and aggression evoking potential of the MSSS itself, so without bringing participants in a state of anger prior to the test. This may be referred to as an ‘emotion follows action’ design.

In a clinical study, Shafir et al. [42] identified specific movement characteristics that are associated with basic emotions. Feeling angry was predicted by advancing with a strong, sudden and direct effort. Shafir et al. referred to anger as an approach emotion, and a punching movement, which is a universal expression of anger. They also referred to studies showing that angry movements include shaking the fists and stamping the feet, leaning forward, and stretching the arms forward, which can all be described as movements during which the shape of the body advances. Further, anger often produces changes in respiration and increases in muscle tension, which influences voice amplitude and pitch [11–14]. Literature supports that besides bodily expressions the voice too conveys emotions, independent of verbal content. Vocal bursts, such as laughing, growling, and shouting, were found to be a cross-cultural modality of emotional communication [43].

Referring to research on sport performance, Davis et al. [9] found support for the moderating role of anger experience (trait anger) and anger regulation in performance of a gross muscular peak force task (kicking). They plea for ecologically valid studies to explore whether emotion regulation strategies, such as suppression, influence physical performance under contextual variations including anger. Personality variables such as neuroticism and extraversion may guide anger suppression (AI) and expression (AO) [1,9]. In real-life situations, expected consequences of anger-related behaviours may also be of key importance in their regulation [44]. Further, systems of emotional responding may moderate the anger performance relationship, particularly the behavioural inhibition system (BIS) which regulates affect and behaviour in response to threatening signals [7].

The MSSS measures body behaviour. Emotional experiences and cognitive appraisals feed the tendency to perform or to inhibit this behaviour. Coombes et al. [7] refer to the premise of ‘action readiness’ to demonstrate that dispositional differences in behavioural inhibition interact with emotional state to alter force production. This premise originates from Frijda, Kuipers & Ter Schure [45]. They describe anger as antagonistic action, leading to different types of interaction, like moving away from a target, or a movement of ‘going against’. According to Frijda & Parrott [46] emotions basically involve a readiness to act, an ‘ur-emotion’. Action readiness is the motive state that underlies feelings of emotional urge or action tendencies. The concept of action readiness may be related to the body-felt ‘urge to act or shout’ which supports the rationale of the MSSS. A change in action readiness may be triggered by imagination, by stimuli (for example photographs), or by self-produced movements [47]. This notion underlies our present test design of ‘emotion follows action’. Neural evidence for action readiness is the involvement of neurotransmitters, such as vasopressin which subserves a ‘power dominance drive motivation’ that may be linked to antagonism [46].

To have an additional reference for the Shout subtest, though in our set-up included as a measure for body force, we may also refer to research on verbally aggressive behaviour: to curse, scold, and shout [48]. Trait inhibition appeared to be negatively correlated with AO and positively with CAO. Verbal inhibition can occur on the level of anger feeling, anger tendency (wanting but not doing), and aggressive behaviour (doing). Smits & Kuppens [41] found that AO, verbal, and physical aggression were negatively related to BIS and positively to BAS. AI, in turn, was found to be characterized by the opposite pattern.

Within mental health care, motion driven changes in action readiness have been demonstrated across a range of psychiatric disorders, for example: agitation and psychomotor retardation in major depression [7]. In the Netherlands, psychomotor therapy (PMT) uses the MSSS as a therapeutic tool for evaluation as well as for treatment of aggression regulation. Aggression is seen as the behavioural outcome of anger-related feelings, cognitions and impulses. The objective is to overcome fear or guilt and to regulate anger and aggression in a prosocial and self-empowering way. A *‘digital thermometer’* is part of the MSSS software and can be displayed on a computer screen to indicate the level of force production, serving as a feedback-loop for learning to control aggression by gradually increasing and decreasing body expression.

Reviews of emotion regulation have been limited by their focus on self-report measures of affect [49]. In this light, the MSSS aims to contribute to performance-based measurement. An advantage of the MSSS is the inclusion of voice expression, perhaps the most relational indicator of emotion regulation. This is for instance shown by the activation patterns in the amygdala when one is confronted with loud angry voices [50]. This first MSSS validity study particularly draws attention on the role of voice expression in discriminating between anger coping styles.

### Limitations

The MSSS is a newly developed, custom-made performance-based measuring instrument. This is the first explorative study of the MSSS in a select student sample. Some of the procedures need to be refined to avoid as much disturbance as possible, for instance due to differences in shoe wear for the Stamp test, or to differences in punching zone used in the Strike test. The Stamp subtest is the most stable instrument. Regarding the Strike subtest it is hard to say whether the mean force levels and the within subject correlations are inherent in the produced force, or were also influenced by how the bag swings after the first stroke. The bag could be replaced by a free-standing bag on a base filled with water or sand to improve the stability. Such adaptations may enhance the reliability of measurements. The software for analysing the recorded test results will be further simplified in order to enhance the usability in practice. The same holds true for the parameters to be considered. Considering this study being a first exploration, we focused on both force production at specific levels and calculated aggregated outcomes for total force production and its rate and distribution over levels. These outcomes have in general good test-retest reliability. However, the low test-retest reliability of especially the quadratic contrast scores showed their sensitivity for, possibly random, variation. Although they have certain face validity, the low correlations of these specific contrast scores with the direct outcomes and the rare associations with anger control ask for further discussion of their meaning and a possibly new selection and redesign of these measures.

A categorical limitation of our study may be the use of a self-report questionnaire as a reference for test validation of a behavioural measure. However, since both measures represent different dimensions of approaching anger coping, there needs to be a common direction if they are expected to deal with the same questions. The SECS was a suitable choice for its good psychometric properties: the subscales closely match with our research question, and the content of the questionnaire is well-known in international literature as the SECS is derived from the widely used STAXI [33]. For these reasons it makes sense to compare our findings to those obtained with this questionnaire. To further support cross-validation of the MSSS, alternative physiological measures like heart rate variability may be used additionally.

In this study the outcomes are controlled for various participants characteristics. Still, besides anger coping, differences in affordance (perception of the function of an object), self-efficacy, exercise sound judgement, and motor skills, may have influenced force production. Further, the women in this study scored much higher on anger control as compared with the scores of the general population. The number of women with higher scores on anger internalization than externalization may have contributed to the discriminatory power of the MSSS. The high mean score could also refer to a larger effect of self-report bias in this group. Social desirability has been found to be associated with lower reported anger expression and higher reported anger control, as measured using common psychometric instruments like the STAXI [51]. The test performance could also be influenced by the momentary circumstances, for example the laboratory context at the university, including a possible observer bias during the test.

In our test design, no information was gathered on time between stamps, shouts and strikes. Since then it has been suggested that indeed it can be relevant to measure the speed of the performance as it may be associated with anger regulation (an urge to act rapidly, or rather a hesitation to act). In future test designs timing in relation to anger could be taken into account.

A methodological consideration is that the motor behaviours must be captured while the neurological processes are active, that is, during felt emotion [52]. In the current laboratory set-up, the anger-performance relationship was based on brief, structured routines at each force level of the pyramid. So there was perhaps little time to engage in an emotional experience.

To explore differences between groups, we also tried to analyse three comparable groups, respectively scoring high, middle or low on anger coping styles (AI, AO, CAI and CAO). However, the extreme groups appeared to be four times smaller than the middle group of the sample, and were too small to draw conclusions. Still, the trend in differences between the three groups showed consistent patterns in the direction of correlations and underscored our finding of the most significant differences in the Shout subtest.

The moderate inter-correlations between the Stamp, Strike and Shout subtests indicated, that these subtests may not be measuring the same aspects and thus may be of complementary value. Mauss & Robinson [2] notice that experiential, physiological and behavioural responses represent different sources of variance, which limits convergence across measures. For now, it is too early to draw conclusions on the question whether the subtests of the MSSS converge into a consistent measure of anger and aggression regulation.

### Conclusion

This first explorative investigation of the MSSS showed excellent internal consistency of the three subtests and high test-retest reliability. The experiences in the laboratory setting and the relatively low amount of not interpretable data indicated that implementation was feasible. The MSSS was well accepted by participants.

The explorative study to the test validity of the MSSS shows a trend that increasing and decreasing force production correlates with anger coping style in a sample of college students, mainly in performing the Shout subtest and most clearly observable in the performance by women who scored high on anger control and low on anger externalization.

The next step is to find out how correlations between anger coping and body behaviour vary in clinical samples. Also, an ‘action follows emotion’ design will be applied by offering anger provoking stimuli prior to the performance to find out whether and how anger stimuli influence force production and control in performing the MSSS.

The MSSS will be further tested to explore its feasibility for clinical and research purposes. Assessment under various conditions and in various populations is necessary to improve standardization of the MSSS. We feel that our initial findings show that there is potential for this method as both a diagnostic and treatment instrument.

## Supporting information

S1 Data SPSS-output containing original data from the study. (SAV)

## References

[1] Böddeker I, Stemmler G. Who responds how and when to anger? The assessment of actual anger response styles and their relation to personality. Cogn Emot. 2000;14: 737–762.

[2] Mauss IB, Robinson MD. Measures of emotion: A review. Cogn Emot. 2009;23: 209–237. 10.1080/02699930802204677 19809584 PMC2756702

[3] Darwin C. The expression of the emotions in man and animals: University of Chicago Press; 1965.

[4] Frijda NH. The emotions. Cambridge, England: Cambridge University Press; 1986.

[5] Tracy JL, Randles D, Steckler CM. The nonverbal communication of emotions. Curr Opin Behav Sci. 2015;3: 25–30.

[6] Beatty GF, Fawver B, Hancock GM, Janelle CM. Regulating emotions uniquely modifies reaction time, rate of force production, and accuracy of a goal-directed motor action. Hum Mov Sci. 2014;33: 1–13. 10.1016/j.humov.2013.12.001 24576703

[7] Coombes SA, Naugle KM, Barnes RT, Cauraugh JH, Janelle CM. Emotional reactivity and force control: The influence of behavioral inhibition. Hum Mov Sci. 2011;30: 1052–1061. 10.1016/j.humov.2010.10.009 21745694

[8] Coombes SA, Gamble KM, Cauraugh JH, Janelle CM. Emotional states alter force control during a feedback occluded motor task. Emotion. 2008;8: 104–113. 10.1037/1528-3542.8.1.104 18266520

[9] Davis PA, Woodman T, Callow N. Better out than in: The influence of anger regulation on physical performance. Pers Individ Dif. 2010;49: 457–460.

[10] Garfinkel SN, Zorab E, Navaratnam N, Engels M, Mallorquí-Bagué N, Minati L, et al Anger in brain and body: the neural and physiological perturbation of decision-making by emotion. Soc Cogn Affect Neurosci. 2015;11: 150–158. 10.1093/scan/nsv099 26253525 PMC4692323

[11] Juslin PN, Scherer KR. Vocal expression of affect In: Harrigan J, Rosenthal R, Scherer K, editors. The new handbook of methods in nonverbal behavior research. New York: Oxford University Press; 2005 pp. 65–135.

[12] Owren MJ, Bachorowski J. Measuring emotion-related vocal acoustics In: Coan J, Allen J, editors. Handbook of emotion elicitation and assessment. New York: Oxford University Press; 2007 pp. 239–266.

[13] Bachorowski J. Vocal expression and perception of emotion. Curr Dir Psychol Sci. 1999;8: 53–57.

[14] Johnstone T, Scherer KR. Vocal communication of emotion In: Lewis M, Haviland-Jones J, editors. Handbook of emotions. New York: Guilford Press; 2000 pp. 220–235.

[15] Trnka R, Stuchlikova I. Anger coping strategies and anger regulation In: Trnka R, Balcar K, Kuska M, editors. Re-constructing emotional spaces: From experience to regulation. Prague: College of Psychosocial Studies Press; 2011 pp. 89–103.

[16] Breakwell GM. Coping with aggressive behaviour. Leicester: British Psychological Service; 1997.

[17] Probst M, Knapen J, Poot G, Vancampfort D. Psychomotor therapy and psychiatry: what is in a name. Open Complement Med J. 2010;2: 105–113.

[18] Hoek HW. Review of the worldwide epidemiology of eating disorders. Curr Opin Psychiatry 2016;29: 336–9. 10.1097/YCO.0000000000000282 27608181

[19] Hoek HW, New developments in the treatment of eating disorders. Current Opinion in Psychiatry 2015;28: 445–47. 10.1097/YCO.0000000000000196 26382167

[20] Boerhout C, Swart M, Van Busschbach JT, Hoek HW. Effect of aggression regulation on eating disorder pathology: RCT of a brief body and movement oriented intervention. Eur Eat Disord Rev. 2016;24: 114–121. 10.1002/erv.2429 26679955

[21] Boerhout C, Swart M, Voskamp M, Troquete NA, Busschbach JT, Hoek HW. Aggression Regulation in Day Treatment of Eating Disorders: Two‐Centre RCT of a Brief Body and Movement‐Oriented Intervention. Eur Eat Disord Rev. 2017;25: 52–59. 10.1002/erv.2491 27862660

[22] Girodet P, Vaslin P, Dabonneville M, Lacouture P. Two-dimensional kinematic and dynamic analysis of a karate straight punch. Comput Methods Biomech Biomed Engin. 2005;8: 117–118.

[23] Bolander RP, Neto OP, Bir CA. The effects of height and distance on the force production and acceleration in martial arts strikes. J Sports Sci Med. 2009;8: 47–52. 24474886 PMC3879635

[24] Mack J, Stojsih S, Sherman D, Dau N, Bir C. Amateur boxer biomechanics and punch force. In: Jensen, R Ebben, W Petushek, W Richter, C Roemer, K. 28 International Conference on Biomechanics in Sports. Detroit, Michigan, USA; Wayne State University, Biomedical Engineering Department; 2010;1.

[25] Wooster M, Vock CA, Youngs P, Larkin A. Real Time Boxing Sports Meter and Associated Methods. Real time boxing sports meter and associated methods. Patent 6,611,782, Washington, DC: U.S. Patent and Trademark Office. 2003.

[26] Klapman M. Boxing glove accelerometer. Boxing glove accelerometer. Patent 5,723,786, Washington, DC: U.S. Patent and Trademark Office. 1998.

[27] Yamagishi M, Miyaji C. Impulse force estimating device, impulse force estimating method, and medium storing impulse force estimation program. Patent 6,397,151, Washington, DC: U.S. Patent and Trademark Office. 2002.

[28] Liu C, Fujimoto Y, Tanaka Y. Flexible Impact Force Sensor. J Sens Technol. 2014;4: 66–80.

[29] Buśko K, Staniak Z, Łach P, Mazur-Różycka J, Michalski R, Górski M. Comparison of two boxing training simulators. Biomed Hum Kinetics. 2014;6: 135–141.

[30] Neto OP, Silva JH, Marzullo de Miranda, Carolina Ana, Bolander RP, Bir CA. The effect of hand dominance on martial arts strikes. Hum Mov Sci. 2012;31: 824–833. 10.1016/j.humov.2011.07.016 22047701 PMC3274566

[31] Golriz S, Hebert JJ, Foreman KB, Walker BF. The reliability of a portable clinical force plate used for the assessment of static postural control: repeated measures reliability study. Chiropr Man Therap. 2012;20: 1–6. 10.1186/2045-709X-20-1 22620678 PMC3502132

[32] Davis CS. Statistical methods for the analysis of repeated measurements. New York: Springer; 2002.

[33] Van Elderen T, Maes S, van der Kamp L, van der Ploeg H, Ensink J, Spielberger C. Handleiding bij de Zelf-Expressie en Controle vragenlijst. [Manual to the Self-Expression and Control Scale; SECS]. Leiden: Leiden University; 1994.

[34] Spielberger CD, Krasner SS, Solomon EP. The experience, expression, and control of anger In: Janisse P, editor. Individual differences, stress, and health psychology. New York: Springer; 1988 pp. 89–108.

[35] van Elderen T, Verkes R, Arkesteijn J, Komproe I. Psychometric characteristics of the self-expression and control scale in a sample of recurrent suicide attempters. Pers Individ Dif. 1996;21: 489–496.

[36] Van Elderen T, Maes S, Komproe I, Kamp L. The development of an anger expression and control scale. Br J Health Psychol. 1997;2: 269–281.

[37] Cohen J. Statistical power analysis for the behavioral sciences. Hilsdale, NJ: Lawrence Earlbaum Associates 1988.

[38] Hurlbert SH, Lombardi CM. Final collapse of the Neyman‐Pearson decision theoretic framework and rise of the neoFisherian. Ann Zool Fenn. 2009;46: 311–349.

[39] Kensinger EA. Remembering emotional experiences: The contribution of valence and arousal. Rev Neurosci. 2004;15: 241–252. 15526549 10.1515/revneuro.2004.15.4.241

[40] Moir G, Sanders R, Button C, Glaister M. The influence of familiarization on the reliability of force variables measured during unloaded and loaded vertical jumps. J Strength Cond Res. 2005;19: 140–145. 10.1519/14803.1 15705024

[41] Smits DJ, Kuppens P. The relations between anger, coping with anger, and aggression, and the BIS/BAS system. Pers Individ Dif. 2005;39: 783–793.

[42] Shafir T, Tsachor RP, Welch KB. Emotion Regulation through Movement: Unique Sets of Movement Characteristics are Associated with and Enhance Basic Emotions. Front Psychol. 2016;6: 2030 10.3389/fpsyg.2015.02030 26793147 PMC4707271

[43] Simon-Thomas ER, Keltner DJ, Sauter D, Sinicropi-Yao L, Abramson A. The voice conveys specific emotions: evidence from vocal burst displays. Emotion. 2009;9: 838–846. 10.1037/a0017810 20001126

[44] Van Coillie H, Van Mechelen I. Expected consequences of anger‐related behaviours. Eur J Pers. 2006;20: 137–154.

[45] Frijda NH, Kuipers P, Ter Schure E. Relations among emotion, appraisal, and emotional action readiness. J Pers Soc Psychol. 1989;57: 212–228.

[46] Frijda NH, Parrott WG. Basic emotions or ur-emotions? Emot Rev. 2011;3: 406–415.

[47] Smits DJ, De Boeck P, Vansteelandt K. The inhibition of verbally aggressive behaviour. Eur J Pers. 2004;18: 537–555.

[48] Smits DJ, De Boeck P. From anger to verbal aggression: Inhibition at different levels. Pers Individ Dif. 2007;43: 47–57.

[49] Webb TL, Miles E, Sheeran P. Dealing with feeling: a meta-analysis of the effectiveness of strategies derived from the process model of emotion regulation. Psychol Bull. 2012;138: 775–808. 10.1037/a0027600 22582737

[50] Simon D, Becker M, Mothes-Lasch M, Miltner WH, Straube T. Loud and angry: sound intensity modulates amygdala activation to angry voices in social anxiety disorder. Soc Cogn Affect Neurosci. 2017;12: 409–416. 10.1093/scan/nsw131 27651541 PMC5390751

[51] McEwan TE, Davis MR, MacKenzie R, Mullen PE. The effects of social desirability response bias on STAXI‐2 profiles in a clinical forensic sample. Br J Clin Psychol. 2009;48: 431–436. 10.1348/014466509X454886 19555524

[52] Crane E, Gross M. Methodological Considerations for Quantifying Emotionally Expressive Movement Style. Ann Arbor. 2007;1001: 48109–42013.

